# Site-Specific Albumin-Selective Ligation to Human Serum Albumin under Physiological Conditions

**DOI:** 10.1021/acs.bioconjchem.2c00361

**Published:** 2022-11-09

**Authors:** Xingjian Yu, Ming Ruan, Yongheng Wang, Audrey Nguyen, Wenwu Xiao, Yousif Ajena, Lucas N. Solano, Ruiwu Liu, Kit S Lam

**Affiliations:** † Department of Chemistry, University of California Davis, Davis, 95616California, United States; ‡ Department of Biochemistry & Molecular Medicine, School of Medicine, University of California Davis, Sacramento, California95817, United States; § School of Food Science, Nanjing Xiaozhuang University, Nanjing, 211171, Jiangsu, China; ∥ Department of Biomedical Engineering, University of California Davis, Davis, California95616, United States

## Abstract

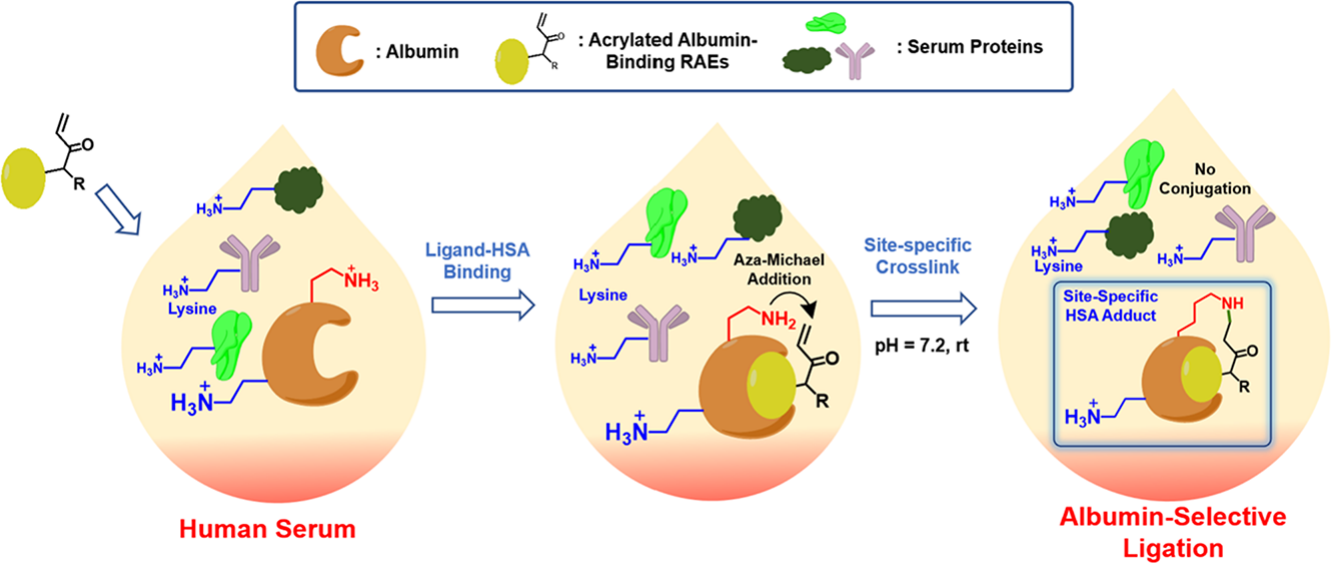

Human serum albumin (HSA) is the most abundant protein in human blood plasma. It plays a critical role in the native transportation of numerous drugs, metabolites, nutrients, and small molecules. HSA has been successfully used clinically as a noncovalent carrier for insulin (e.g., Levemir), GLP-1 (e.g., Liraglutide), and paclitaxel (e.g., Abraxane). Site-specific bioconjugation strategies for HSA only would greatly expand its role as the biocompatible, non-toxic platform for theranostics purposes. Using the enabling one-bead one-compound (OBOC) technology, we generated combinatorial peptide libraries containing myristic acid, a well-known binder to HSA at Sudlow I and II binding pockets, and an acrylamide. We then used HSA as a probe to screen the OBOC myristylated peptide libraries for reactive affinity elements (RAEs) that can specifically and covalently ligate to the lysine residue at the proximity of these pockets. Several RAEs have been identified and confirmed to be able to conjugate to HSA covalently. The conjugation can occur at physiological pH and proceed with a high yield within 1 h at room temperature. Tryptic peptide profiling of derivatized HSA has revealed two lysine residues (K225 and K414) as the conjugation sites, which is much more specific than the conventional lysine labeling strategy with *N*-hydroxysuccinimide ester. The RAE-driven site-specific ligation to HSA was found to occur even in the presence of other prevalent blood proteins such as immunoglobulin or whole serum. Furthermore, these RAEs are orthogonal to the maleimide-based conjugation strategy for Cys34 of HSA. Together, these attributes make the RAEs the promising leads to further develop *in vitro* and *in vivo* HSA bioconjugation strategies for numerous biomedical applications.

## Introduction

Human serum albumin (HSA) is the most abundant protein in human blood plasma.^26^ HSA has been well established as a platform for various diagnostic and therapeutic applications.^1^ These include HSA being successfully used clinically as a non-covalent carrier for insulin (e.g., Levemir), GLP-1 (e.g., Liraglutide), and paclitaxel (e.g., Abraxane).^2^ Efforts have been made to use HSA as a covalent carrier for drug delivery. However, none has been approved for clinical use so far. The development of post-translational chemical modifications that can derivatize native HSA site-specifically under mild reactions will allow researchers to engineer HSA-drug conjugates or supramolecular HSA-based nanostructures with desirable pharmacokinetic (PK)/pharmacodynamic (PD) properties and protein–adduct ratio, for various biomedical applications. The HSA’s only free cysteine (Cys34) makes maleimide chemistry a viable approach to site-specifically modify HSA.^3^ One of the concerns for the maleimide-based conjugation strategy, however, is that the resulting thiosuccinimide linkage is unstable through the retro-Michael reaction,^4^ or thiol exchange, which poses risks in the performance and safety of the HSA conjugates due to possible unexpected payload release. Furthermore, for targeted drug delivery using HSA as the carrier, it would be advantageous to have additional site-specific ligation strategies that are orthogonal to traditional maleimide chemistries, such that targeting ligands and one or more payloads can be reliably and site-specifically conjugated to HSA to form a homogeneous conjugate.

**Figure 1:**
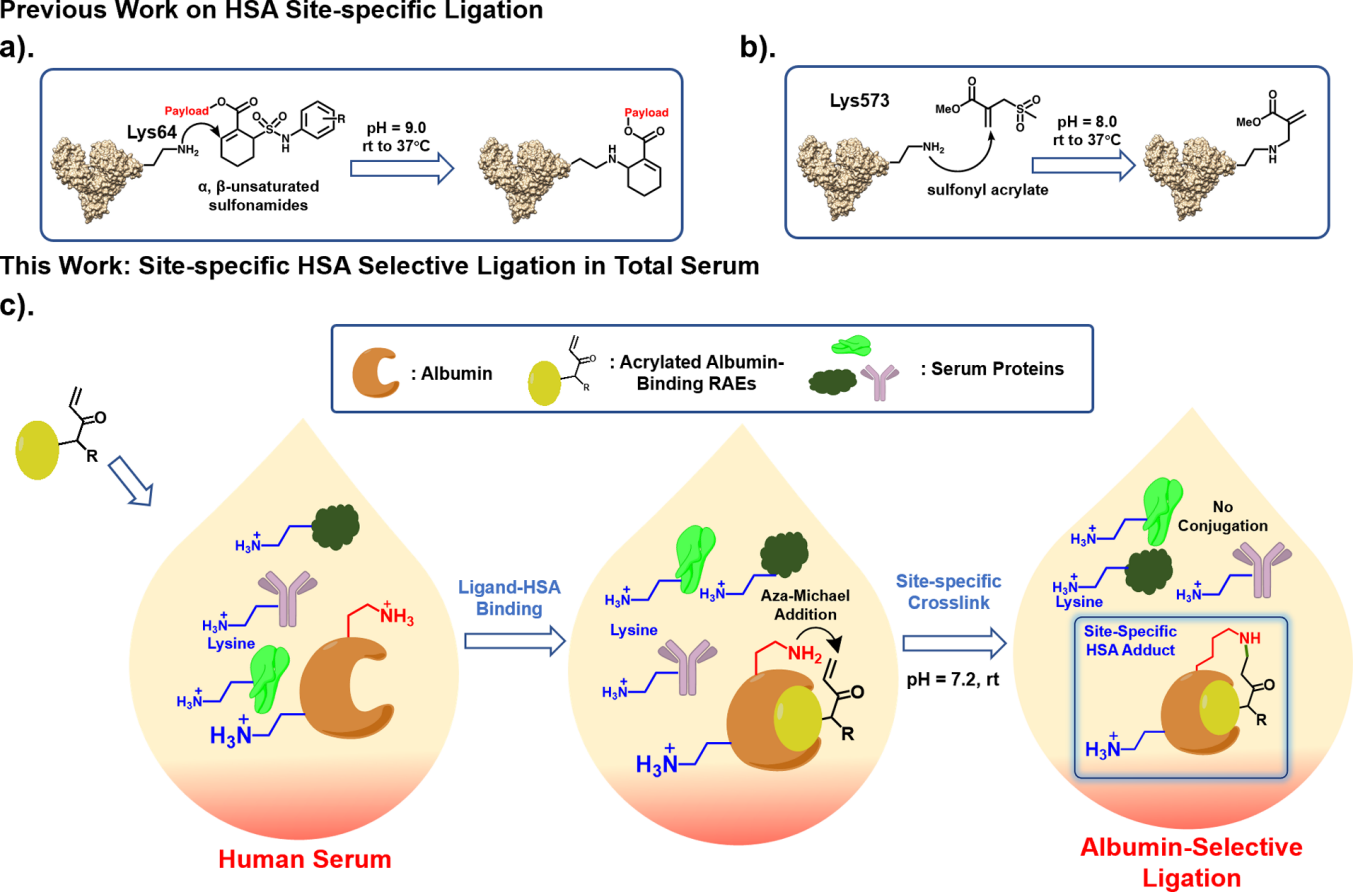
(a) α,β-unsaturated sulfonamides specifically modified K64 of HSA; (b) sulfonyl acrylate specifically modified K573 of HSA; (c) this work: site-specific HSA lysine modification via unactivated acrylamide in the reactive ligand driven by the proximity effect in a complex protein mixture such as human serum.

The ε-amino group on the lysine side chain is another popular site for protein conjugation. The cationic nature of lysine residues at physiological pH makes their distribution relatively common on the protein surface and more accessible to conjugation reagents.^5^ The conventional lysine conjugation strategy is through reaction with electrophiles, such as *N*-hydroxysuccinimide ester (NHS-ester), isothiocyanate, or activated aromatic esters.^6^ Site-specific conjugation to lysine is more challenging as lysines are abundant in the proteome. For example, for HSA, there are 48 lysines in total. These highly reactive electrophiles often fail to differentiate particular lysines, and they tend to react with lysines randomly. Using these non-specific bioconjugation techniques to prepare protein conjugates may compromise the properties of proteins by accidentally labeling physiologically important amino acids. For example, domain IIIB and domain I of HSA are known to be essential for their binding to cell surface FcRn, which is responsible for the recycling of HSA.^7^ Modification of surface lysines on these two HSA domains will likely lead to a decrease in the circulation half-life of HSA. In the case of antibody-drug conjugates (ADC), non-specific bioconjugation techniques involving lysines could bring unexpected cytotoxicity to the protein conjugates,^8^ while on the contrary, homogeneous ADCs prepared by site-specific approaches have demonstrated an improved therapeutic index.^9^ Nevertheless, there have been several studies on performing site-specific conjugation on lysine residues,^10^ and site-specific ligations to HSA utilizing aza-Michael addition between the ε-amine of lysine residues and Michael acceptors, such as α,β-unsaturated sulfonamides and sulfonyl acrylate (Figure 1a,b) have also been reported.^11^ The chemo and regioselectivity of these strategies originate from kinetic control or delicately tuned substrates such that lysine with higher nucleophilicity and better accessibility can react preferentially. These generic bioconjugation methods, however, are reactive to a variety of proteins. For *in vivo* applications, it is demanding to develop HSA-exclusive methods to avoid off-target ligation.

Inspired by the design of selective covalent drugs targeted on lysine residues,^12^ we set out to develop a robust ligation strategy to prepare a chemically well-defined HSA conjugate, such that ligation can occur *in vitro* or *in vivo* under physiological conditions. To achieve this, it will be necessary to (1) reduce the overall electrophilicity of Michael acceptor to lower the chances of off-target conjugation and (2) conditionally “turn on” the nucleophilicity of particular lysine(s) on HSA. Unactivated α,β-unsaturated acrylamide or acrylate ester lacks electrophilicity, so nucleophilic attack from biological amine and thiol to these Michael acceptors is generally difficult under physiological conditions.^13^ Therefore, these moieties have been integrated into many covalent inhibitor designs to minimize off-target labeling.^14^ The p*K*_a_ of polarizable amino groups within a protein can vary greatly (up to ∼100,000 fold) depending on the nanoenvironment around these residues.^15^ Similarly, many enzymes can be activated/deactivated upon ligand/substrate-protein binding, as it can alter the nanoenvironment of crucial amino acid residues at active sites.^16^ Herein, we hypothesize that a molecule containing a known affinity binder of HSA and unactivated acrylate will specifically bind to a particular binding pocket to alter the nanoenvironment of the binding pocket, which promotes the nucleophilicity of lysine in proximity and makes it reactive enough for unactivated acrylate in the binding molecule or what we call “reactive affinity element” (RAE). In principle, these RAEs will specifically derivatize the lysine at the proximity of the binding pocket but will stay inert to other biological nucleophiles displayed on HSA or other blood proteins.

Related proximity ligation strategies have been exploited to chemically derivatize other proteins site-specifically. For example, Kishimoto et al.^17^ reported using activated NHS ester derivatized Fc affinity peptides to selectively conjugate to Lys248 of human IgG Fc. This strategy demonstrates chemical selectivity toward IgG in the presence of IgA and HSA. However, part of the observed selectivity might be the result of kinetic control as the conjugation reaction was carried out for only 15 min at dilute protein concentration (1.9 μM), and it is unclear if the Fc affinity peptides can alter the p*K*_a_ of Lys248. To the best of our knowledge, there is no precedence to locally augment the nucleophilicity of particular lysine on target proteins such that it can react with unactivated acrylate or acrylamide.

**Figure 2:**
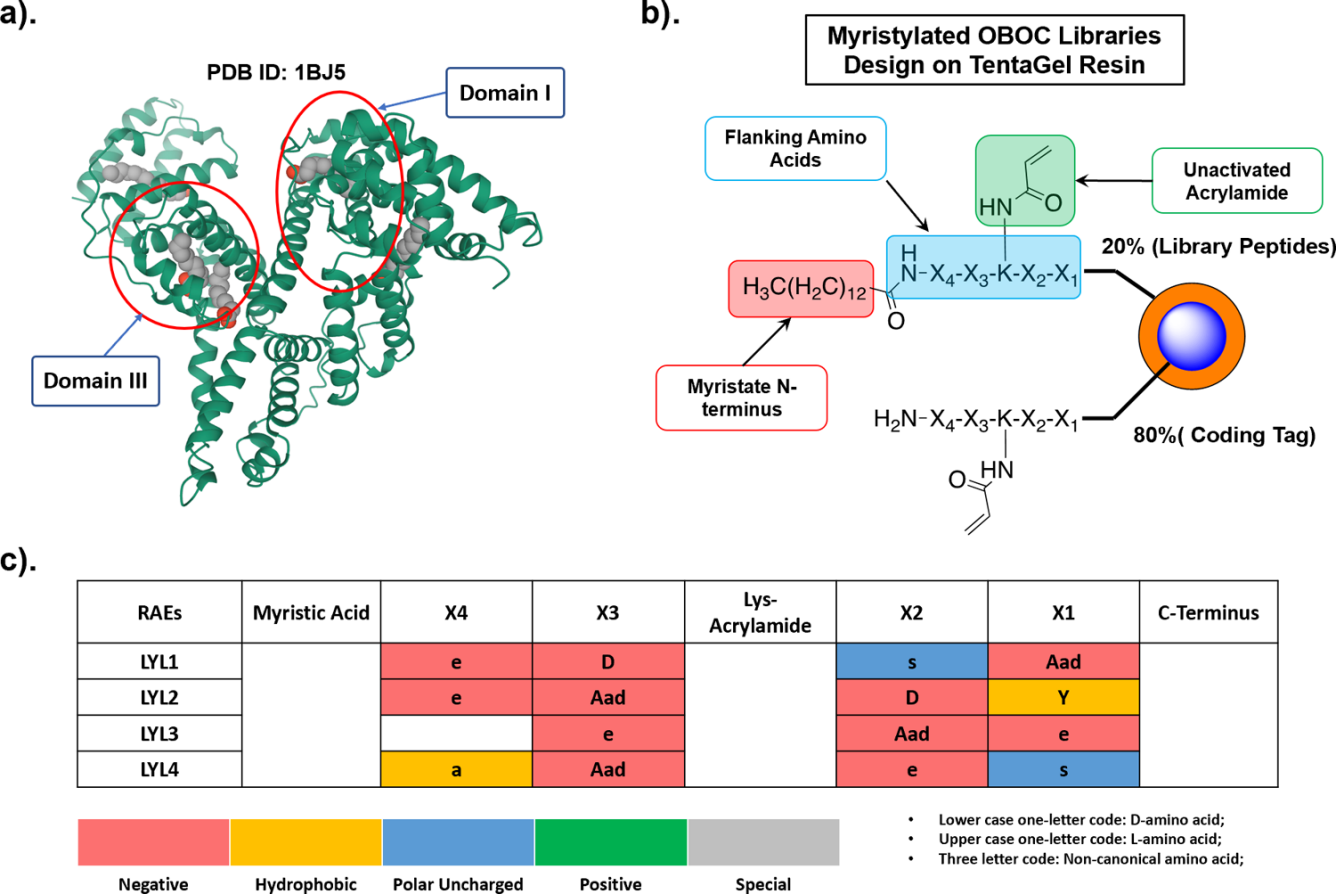
(a) Published X-ray diffraction studies reveal 5 hydrophobic pockets where myristic acid can bind (gray balls).^18^ Myristic acid can bind to domains I and III (red circle) more strongly; (b) design of OBOC libraries. The library compounds comprised 3 parts: myristylated N-terminus (red), random peptides made by 36 different amino acids (blue), and unactivated acrylamide branched from the side chain of a lysine residue (green); (c) sequence of discovered RAEs (LYL1–4) that are reactive toward HSA.

We previously reported the use of the one-bead one-compound (OBOC) combinatorial library method^19^ to discover several indole-based fluoro-dinitrophenyl peptidomimetic ligands that bind to and chemically react with the Fab domain of the immunoglobulin.^20^ In this study, we leverage the well-known ligands to HSA^21^ to design focused OBOC combinatorial peptide libraries to discover RAEs that can site-specifically conjugate to HSA via unactivated acrylamide. We chose myristic acid, a 14-carbon long-chain fatty acid, as the affinity ligand for proximity covalent ligation. X-ray diffraction identified five myristic acid/myristate binding sites on HSA; therein, the interaction at domain I and domain III is much stronger, with the binding affinity ranging between 50 nM to 1 μM (Figure 2a).^18,22^ We used the enzyme-linked immuno-staining approach to screen the OBOC libraries and identified four myristylated peptidomimetic RAEs that can covalently modify HSA through specific lysine(s) under mild physiological pH conditions. The most reactive RAE, LYL1, has demonstrated site-specificity in the vicinity of myristate binding pockets, with selectivity to HSA over other serum proteins. It also showed excellent compatibility with different lysine-specific and cysteine-specific protein modification strategies.

## Results and Discussion

### OBOC Library Design & Screening

We prepared three random myristylated OBOC peptidomimetic libraries, Myr-K(Acryl)X_2_X_1_-bead, Myr-X_3_K(Acryl)X_2_X_1_-bead, and Myr-X_4_X_3_K(Acryl)X_2_X_1_-bead, which contain 2-, 3-, and 4-diversity, respectively (Figures S1–S3), with a total diversity of ∼1.6 million (Figure 2b). Myristic acid was used to cap the N-terminus of peptides in the libraries through 6-Cl HOBt/DIC coupling. Although longer fatty acids, such as palmitic acid (*n* = 16) and stearic acid (*n* = 18), bind tighter to HSA,^22,23^ the increased hydrophobicity could limit the application in physiological environments. The acrylamide was derived from acrylic acid by reacting with the ε-NH_2_ of a fixed lysine residue protected by Dde (1-(4,4-dimethyl-2,6-dioxocyclohex-1-ylidene)ethyl), a reductive labile protecting group orthogonal to Fmoc (fluorenylmethyloxycarbonyl) chemistry.^24^ This fixed lysine is flanked by random residues comprising 36 canonical and non-canonical amino acids, 50% of which have the d-configuration (Table S1). We diversified the libraries chemically and stereometrically by introducing non-canonical amino acid and d-amino acid to promote the likelihood to find more specific and reactive RAE toward HSA and to make the discovered RAEs more resistant to proteolysis. As a result, the final HSA conjugate may have a longer serum half-life and better pharmacokinetic properties suitable for *in vivo* applications. To ensure that the libraries are decipherable by the Edman degradation sequencer, we used a biphasic-encoding strategy to preserve free N-terminal amines for ∼80% of the peptide substitution in the bead interior as the coding tag.^25^ Of about 1.5 million beads screened (Supporting Information S3), we identified 4 RAEs, LYL1, LYL2, LYL3, and LYL4, with sequences summarized in Figure 2c. The amino acids are predominantly negatively charged [glutamate, aspartate, and 2-aminoadipic acid (Aad)], suggesting that interactions between HSA and RAEs are unique for covalent conjugation.

### Characterization of Conjugation

**Figure 3:**
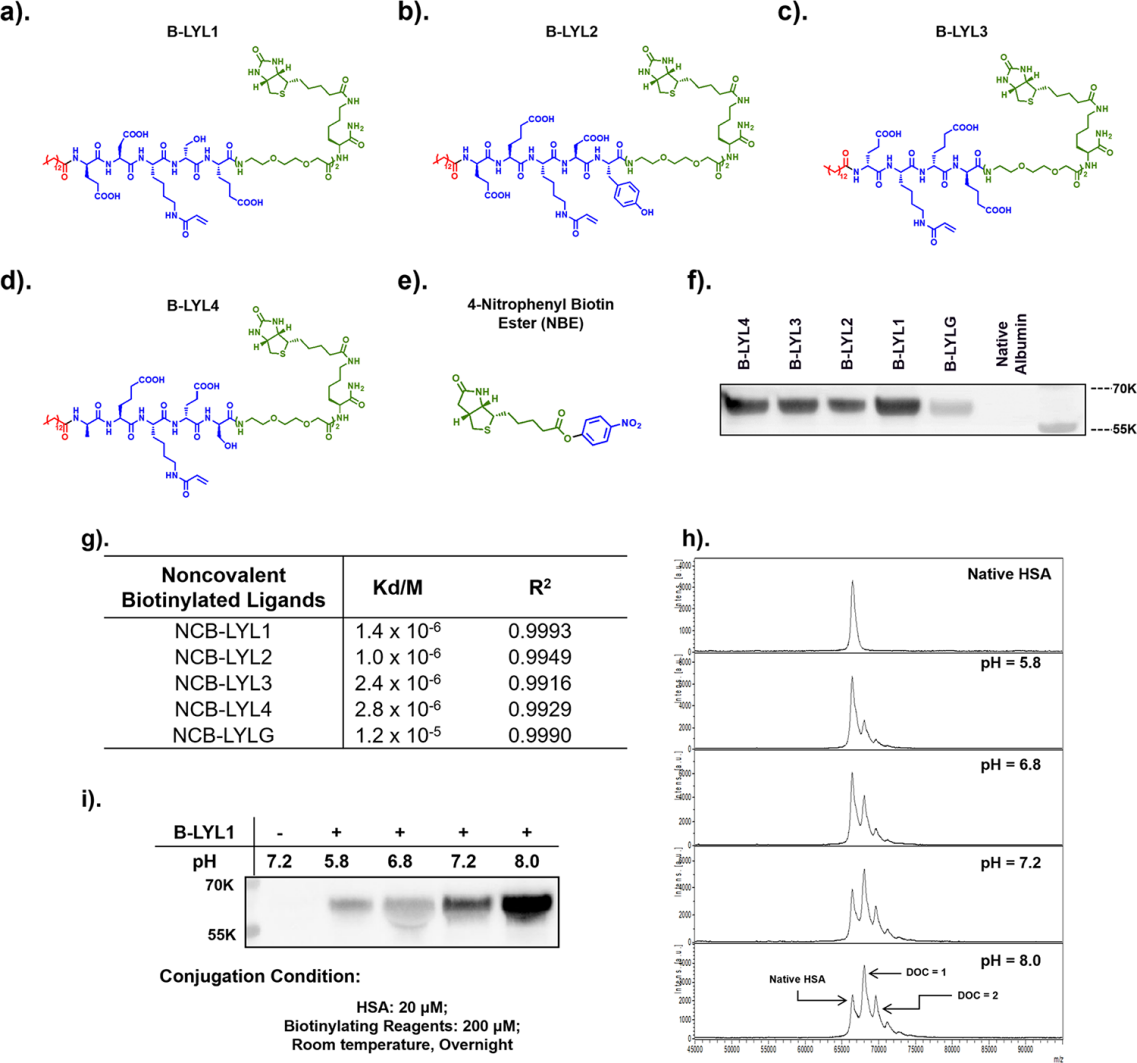
(a–e) Chemical structure of biotin-tagged RAEs LYL1–4 and non-selective 4-nitrophenyl biotin ester (NBE) as positive control used to chemically biotinylate HSA; (f) western blots to detect HSA biotinylated by peptidomimetic RAEs. The conjugation was performed in PBS buffer (pH = 7.2) with 20 μM HSA and 100 μM biotinylation reagents for 1 h at room temperature; (g) bio-layer interferometry assay measured the intrinsic binding affinity using non-covalent biotin-LYLs; (h) MALDI-TOF intact MS for HSA-LYL1BT conjugates prepared at various pH values in PBS. The conjugates were prepared by mixing 20 μM HSA and 200 μM B-LYL1 for 16 h; (i) the intensity of corresponding western blots is proportional to signal intensity from biotinylated HSA in MS and increases as pH elevates.

To confirm the covalent conjugation of RAEs to HSA, the RAEs were tagged with biotin (B-LYL1, B-LYL2, B-LYL3, and B-LYL4, Figure 3a–d, Supporting Information S4) and the final HSA-biotin conjugates were detected by western blotting using a streptavidin Alexa-647 conjugate (SA-647). To minimize possible interference as well as to improve the solubility of RAEs in aqueous buffer, two hydrophilic AEEA (2-aminoethoxy-2-ethoxy acetic acid) linkers were placed between the C-terminus of RAE and Lys(biotin). 4-Nitrophenyl biotin ester (NBE), an activated ester known to non-specifically biotinylate many lysine residues on protein via nucleophilic substitution, was used as the positive control (Figure 3e).^27^

Through western blotting (Figure 3f), we found that all four RAEs could covalently conjugate to HSA at physiological pH of 7.2 within 1 h in PBS buffer. The absence of the signal for native HSA not only suggested minimal albumin-streptavidin interaction but also demonstrated that the possible thiol-Michael addition between the Cys34, the signature free thiol on HSA, and acrylamide was insignificant, suggesting the ligands’ chemoselectivity toward lysine over cysteine. We then investigated how the different molecular features of these RAEs can influence their reactivity toward HSA. When the myristoyl tail was replaced with an acetyl group, all these RAEs lost their reactivity, indicating that the long-chain aliphatic acid at N-terminus is essential for site-specific ligation. We observed a much weaker signal for B-LYL-G where the flanking amino acid residues were replaced by glycine, indicating that these negatively charged amino acids are also important to promote ligation. We also explored the RAEs’ intrinsic non-covalent binding affinity toward HSA and investigated how the negative charges of these peptides can influence the binding affinity and reactivity. We synthesized the non-covalent reacting version of these biotinylated RAEs, NCB-LYL1–4 (Figure S6), by replacing acrylamide with an acetyl group and performed a bio-layer interferometry (BLI) assay to measure the binding strength between HSA and RAEs. According to the BLI assay, these modified RAEs demonstrated modest binding affinity toward HSA with the *K*_d_ values in the range of 1 to 3 μM (Figure 3g and Figure S7), which is comparable with fatty acid-modified insulin^28^ used in the clinic. The counterpart of B-LYL-G, NCB-LYL-G, showed weaker binding affinity at 12 μM relying solely on hydrophobicity, suggesting that the presence of a negatively charged amino acid residue can enhance binding affinity by up to 10-fold. These results indicated that the hydrophobic moiety of RAEs and negative charges from flanking amino acid residues are indispensable to promoting the RAEs’ high reactivity toward HSA. We anticipate that the myristoyl tail of the RAE affords a less polarizable nanoenvironment through desolvation of the targeted lysine, making the deprotonation state (lower p*K*_a_) more favorable and thus becoming more nucleophilic,^15,29^ while the flanking amino acid residues of the RAEs enhance the binding affinity to make the conjugation more efficient and specific.

We also employed a modified neutravidin pull-down assay to measure the conjugation yield quantitatively and systematically studied the parameters that affect conjugation efficiency (Supporting Information S6).^30^ To validate the assay, we determined the yield for conjugations performed under the initial condition. We found that the immobilized neutravidin agarose exhibited little non-specific uptake for underivatized HSA at physiological pH 7.2. In contrast, approximately 50% uptake was observed for the HSA-RAE conjugate and 100% for the NBE conjugate (Figure S10). Next, we examined the conjugation yield at different pHs as aza-Michael addition is more favorable at alkaline pH. As expected, we found that the conjugation yield could be improved to 60%–80% at pH 8.0. We also found that using a higher equivalent of RAEs could promote conjugation yield, and B-LYL1 yielded more than 90% conversion at 10 equivalents to HSA. Prolonging the conjugation time to more than 3 h could also enhance conjugation efficiency (Figures S11–S13).

### Characterization of Site Specificity

According to the neutravidin pull-down assay, B-LYL1 is the most reactive RAE toward HSA. Therefore, we focused our effort on characterizing the interaction between B-LYL1 and HSA. We used a matrix-assisted laser desorption/ionization time-of-flight (MALDI-TOF) mass spectrometer to determine the degree of conjugation (DOC), defined as the number of covalent adducts per HSA molecule. At 10 equivalents of B-LYL1 and 16 h of conjugation, the MALDI-TOF generated consistent results corroborating with the western blot (Figure 3i), where more HSA were chemically conjugated with increasing pH (Figure 3h). The level of bivalent or multivalent HSA conjugates (DOC = 2 or more) was found to elevate with increasing pH causing deprotonation of lysine residues, although monovalent conjugates were predominant at all pH levels.

To identify the ligation sites, we performed tryptic proteomic analysis of recombinant HSA after treatment with B-LYL1. B-LYL1 adducts were found at K225 and K414 containing peptides (Figures S15 and S16), indicating that these two lysines are the sites of covalent modification. Computationally, we profiled the protonation states of lysine residues from HSA.^31^ The results reveal K199 as the most reactive lysine with the lowest p*K*_a_, which follows experimental data (Supporting Information S8 and Table S5).^32^ The selective modification on K225 and K414 over K199 suggests that B-LYL1 can specifically “turn on” the lysines that are chemically inert. Although the relative reactivity of B-LYL1 toward K414 and K225 has yet to be determined, it is clear that B-LYL1 can afford much more homogeneous and chemically well-defined HSA conjugates by ligating to one or two specified lysine residues out of possible 48 lysines in HSA.

### Site-Specific HSA Ligation within the Complex Protein Mixture

**Figure 4:**
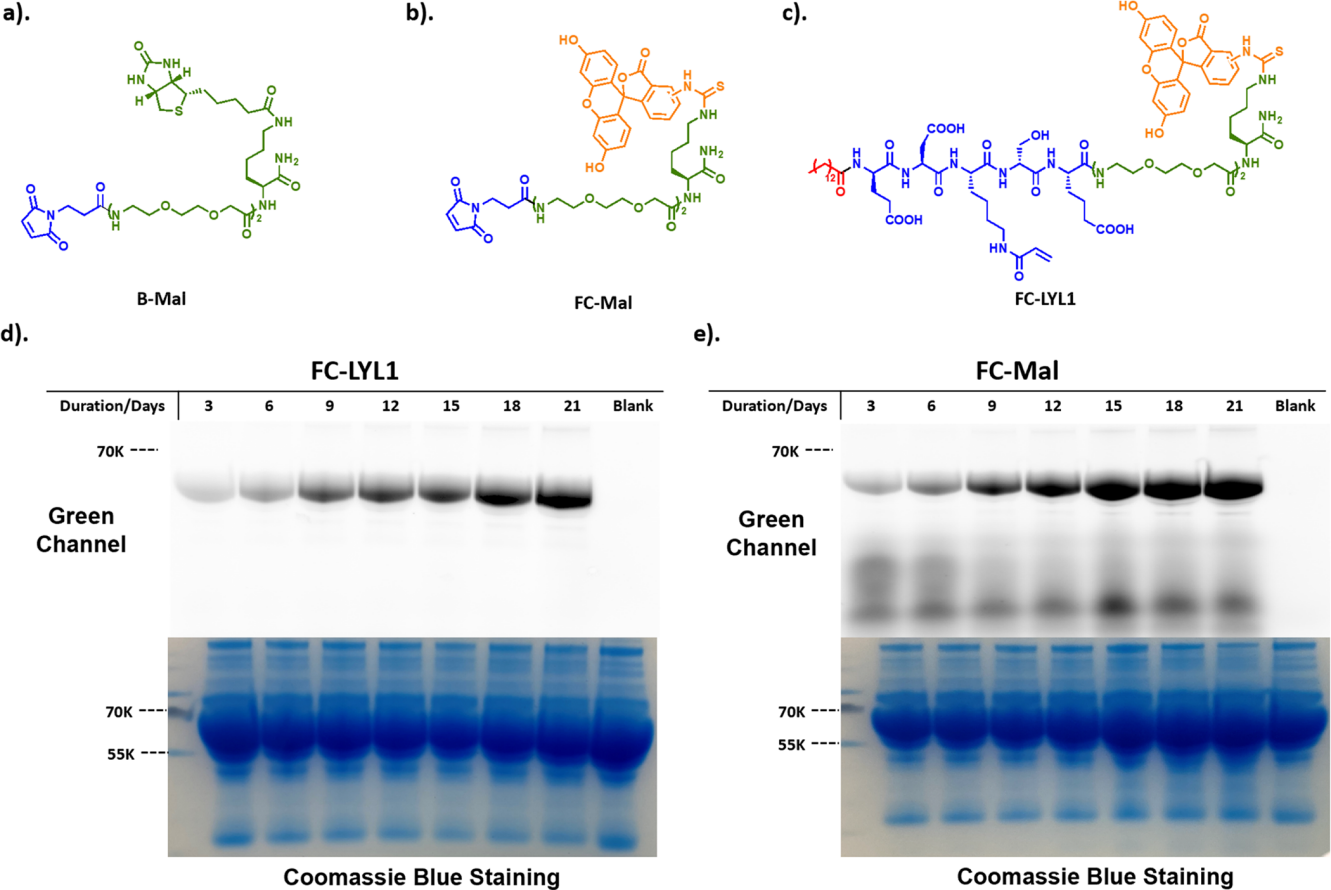
(a–c) Chemical structure of biotin-tagged maleimide (B-Mal), FITC-tagged maleimide (FC-Mal), and FITC-tagged LYL1 (FC-LYL1) used for selective HSA conjugation in a complicated protein matrix; (d, e) comparison between FC-LYL1 and FC-Mal in modifying proteins in serum. Protein SDS-PAGE gels were exposed to a green channel (Ex = 490 nm, Em = 525 nm) and then stained with Coomassie Blue. Electrophoresis showed that FC-LYL1 can selectively label albumin content (d) while FC-Mal labels multiple proteins (e).

To further demonstrate the versatility and robustness of B-LYL1 in site-specific modification of HSA and its advantages over other kinetic-driven site-specific ligation approaches, we investigated if LYL1 can perform selective HSA chemical modification and differentiate other proteins. First, we investigated if B-LYL1 can chemically conjugate to HSA in the presence of human intravenous immunoglobulin (IVIG, clinical-grade polyclonal antibodies isolated from plasma of normal donors),^33^ as a start to study LYL1’s specificity toward HSA. We treated HSA, IVIG, and a HSA/IVIG mixture with B-LYL1 and maleimide-based biotinylation reagent, B-Mal (Figure 4a), at physiological pH (Figure S19). In the HSA/IVIG mixture, we only detected signals from HSA when the mixture was treated with B-LYL1. We were concerned that the acrylamide of B-LYL1 might react with IVIG through the thiol-Michael pathway^34^ since thiols from cysteine are generally better nucleophiles than ε-amine from lysine residues. The absence of signals from the IVIG portion proves that B-LYL1 was specific against HSA and would not biotinylate other proteins via thiol-Michael addition.

Encouraged by the promising selectivity, we then evaluated LYL1’s selectivity in human serum, which is a mixture of many proteins at various levels, over a 3 week time course of incubation at 37 °C. Using biotin as the tag in this experiment is problematic because a significant number of serum proteins are endogenously biotinylated.^35^ Therefore, we synthesized the fluorescein-tagged version of the RAE, fluorescein-LYL1 (FC-LYL1, Figure 4c), and compared its bioconjugation profile with fluorescein-tagged maleimide (FC-Mal, Figure 4b). We found that the acrylamide-based LYL1 was highly selective toward HSA over 3 weeks (Figure 4d), even under conditions (longer time and higher temperature) that could potentially compromise LYL1’s selectivity toward HSA by promoting side reactions with other biological nucleophiles from other proteins. In contrast, FC-Mal not only labeled HSA (Cys34) but also several other serum proteins as expected (Figure 4e). Interestingly, the ligation reaction was found to continue to increase over the first 2 weeks of incubation. In this experiment with whole serum, the conjugation was done in excess HSA. Given that there are 5 known fatty acid binding pockets per HSA molecule, we believe a significant portion of the reactive probes would be sequestered in the “non-productive” (no covalent ligation) fatty acid binding sites most of the time. Consequently, unlike the result shown in Figures S11–S13 and Tables S3 and S4 with fast ligation kinetics and good yield (in the setting of excess LYL1 to HSA), here, we observed slow reaction over days for both probes. Nonetheless, this experiment does confirm that site-specific ligation of albumin in the context of complex human serum can be achieved with the LYL1 probe, albeit taking days to complete. This data on selectivity suggests that LYL1 can potentially derivatize serum albumin site-specifically *in vivo* if administered intravenously. However, to be useful clinically, we may need to further optimize LYL1 for faster ligation kinetics.

### One-Pot Dual-Modification of HSA

**Figure 5:**
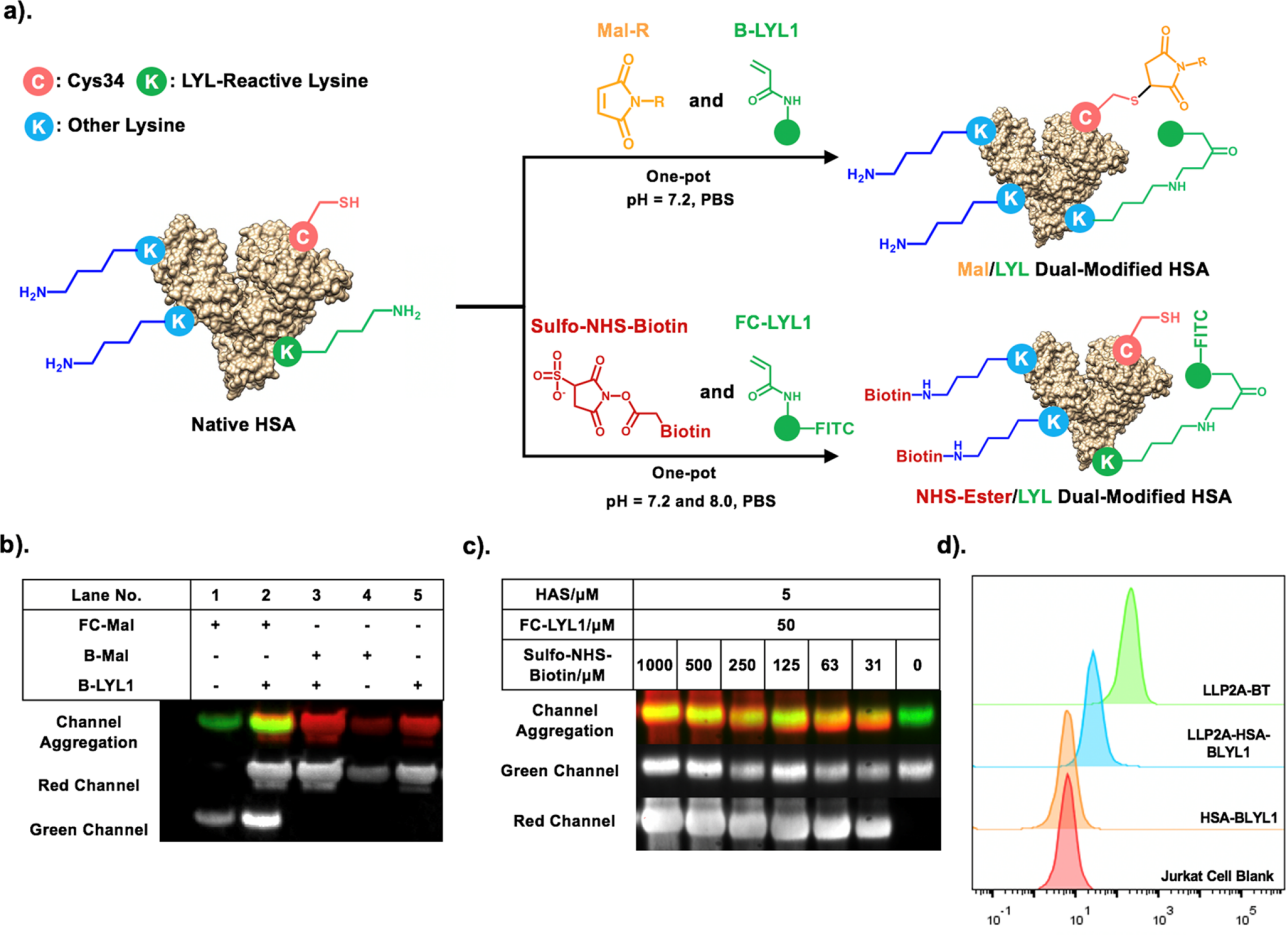
(a) Ability of conjugation to specific lysine allows B-LYL1 to modify HSA concurrently with other conjugation strategies in one pot to afford dual-modified HSA conjugates; (b) western blots for dual-labeled HSA conjugates. B-LYL1 can biotinylate HSA on lysine in the presence of fluorescein-Mal (F-Mal) that modifies cysteine residues. HSA biotinylation was detected by streptavidin Alexa 647 conjugates at the red channel (Ex = 594 nm, Em = 633 nm), while the fluorescein tag was detected at the green channel (Ex = 490 nm, Em = 525 nm). (c) Western blots for dual-labeled HSA conjugates using FC-LYL1 and Sulfo-NHS-Biotin. Dual-conjugation status was examined by similar approaches to (b). (d). Flow cytometry of the LLP2A-HSA-B-LYL1 (20 μM) complex that binds to Jurkat cells. Biotinylated LLP2A ligands (B-LLP2A, 20 μM) were used as the positive control.

The ability to simultaneously and orthogonally introduce multiple molecular moieties into biomolecules would greatly expand the clinical translational scope of the resulting conjugates.^36^ A recent report has demonstrated approaches for dual site-selective protein labeling of lysine and cysteine of HSA, where lysine and cysteine were labeled sequentially at pH 8.0.^37^ LYL1’s high specificity toward K414 and K225 in mild physiological conditions makes us believe that we can simultaneously modify HSA using LYL1 in combination with other chemistries by a one-pot scheme (Figure 5a). To prove the prediction, we performed the one-pot reaction experiment shown in Figure 5a using B-LYL1, FC-Mal, or B-Mal at physiological pH (pH = 7.2) in PBS at room temperature for 1 h, and the fluorescently-labeled ligation products were analyzed by western blotting (Figure 5b).^5,38^ It is clear that dual derivatization of HSA was achieved in one-pot by mixing B-LYL1 simultaneously with either B-Mal or FC-Mal. We also tested LYL1’s compatibility with fluorescein, which is commonly used to tag proteins fluorescently through lysine and cysteine residues.^39^ Under alkaline pH, the reactivity of FITC can be enhanced, which may competitively undermine the site specificity of B-LYL1. Our results indicated that even in the presence of FITC, B-LYL1 was able to label HSA well at pH 7.2 and pH 8.0 (Figure S20). In another experiment, we tested LYL1’s compatibility with the well-known non-selective lysine labeling reagent Sulfo-NHS biotin. The results showed that FC-LYL1 could label HSA even in the presence of 200-fold excess NHS ester (Figure 5c). We further concurrently modified HSA with B-LYL1 and maleimide-derivatized LLP2A (LLP2A-Mal) in a one-pot reaction at physiological pH. LLP2A was previously reported by us as a high-affinity peptidomimetic ligand against activated α4β1 integrin.^40^ Using flow cytometry analysis, we demonstrated that the resulting bifunctional HSA conjugate (biotinylated and LLP2A-derivatized HSA or B-LYL1-HSA-LLP2A) could indeed bind to Jurkat T-leukemia cells overexpressing activated α4β1 integrin (Figure 5d). This result not only confirms the orthogonality of LYL1 and maleimide derivatization of HSA at physiological pH but also demonstrates that targeting HSA–drug conjugates or imaging agents can be prepared by this robust mild conjugation strategy. The resulting conjugate is expected to have a long blood circulation half-life and low immunogenicity.^41^

## Conclusions

We utilized the OBOC combinatorial chemistry to discover four myristate RAEs that can covalently cross-link to HSA through unactivated acrylamide under mild physiological pH conditions. The RAE with the highest conversion, LYL1, can conjugate to K225 and K414 of HSA via aza-Michael addition. With LYL1, HSA ligation can occur efficiently and site-specifically even in complex protein mixtures such as human serum without reacting to other proteins. Furthermore, the LYL1 ligation chemistry is utterly orthogonal to the well-known Cys34 derivatization with maleimide, thus allowing a simple and robust one-pot reaction of dual derivatization. In addition to preparing HSA–drug conjugates, these orthogonal ligation reactions will enable us to develop chemically defined supramolecular HSA nanocarriers for various biomedical applications. Work is currently underway in our laboratory to use computational chemistry to model the various RAEs discovered by the OBOC combinatorial chemistry method. Combining these two powerful but very different chemical methods will allow us to (1) gain important insight into the structure reactivity and structure specificity of the RAEs and (2) design highly focused OBOC libraries for the discovery of novel orthogonal RAEs with faster ligation kinetics, higher plasma stability, and minimal conformational interruption to HSA. We can envision that such improved orthogonal RAEs will enable us to design and synthesize HSA–drug conjugates and well-defined HSA-based nanoplatforms for various biomedical applications. Furthermore, we may also leverage the FcRn on immune cells to deliver immunostimulants and antigenic peptides via such HSA–vaccine conjugates. Although this work focuses on HSA, the same approach can be applied to discovering RAEs for other important human plasma proteins or therapeutic proteins of clinical importance.

## Supporting Information Available

Chemicals and materials; OBOC library design and synthesis; enzymatic colorimetric screening procedures; solid-phase synthesis for RAEs and derivatives; BLI assay for biotinylated noncovalent peptidomimetics; quantification of biotinylated HSA using the neutravidin pull-down assay; tryptic digestion and proteomics analysis; computational prediction for the protonation states of HSA; general procedures to prepare and characterize HSA–RAE conjugates; and MALDI-TOF mass spectrum for molecular characterization (PDF)
