# Supplementary material for: Site-Specific Albumin-Selective Ligation to Human Serum Albumin under Physiological Conditions
Source: Bioconjug Chem. 2022 Nov 9;33(12):2332–40. doi: 10.1021/acs.bioconjchem.2c00361 (PMC9782315; doi:10.1021/acs.bioconjchem.2c00361)
Supplement: Supplementary file 1 — bc2c00361_si_001.pdf [file bc2c00361_si_001.pdf]

# Site-Specific Covalent Ligation of Human Serum Albumin under Physiological Condition

Xingjian Yu,[1,2] Ming Ruan,[2] Yongheng Wang,[2,3] Audrey Nguyen,[2] Wenwu Xiao,[2] Yousif Ajena,[2] Lucas N. Solano,[2] Ruiwu Liu[2*], and Kit S Lam[2*]

1. Department of Chemistry, University of California Davis, Davis, 95616, CA, USA;
2. Department of Biochemistry & Molecular Medicine, School of Medicine, University of California, Davis, Sacramento, CA, 95817, USA;
3. Department of Biomedical Engineering, University of California Davis, Davis, CA 95616, USA;

## *Supporting Information*

# Table of Content

S1. Chemicals & Materials
S2. OBOC Library Design & Synthesis
S3. Enzymatic Colorimetric Screening Procedures
S4. Solid Phase Synthesis for RAEs and Derivatives
S5. BLI Assay for Biotinylated Non-covalent Peptidomimetics.
S6. Quantification of Biotinylated HSA using Neutravidin Pull-Down Assay
S7. Tryptic Digestion and Proteomics Analysis
S8. Computational Prediction for Protonation States of HSA
S9. General Procedures to Prepare and Characterize HSA-RAEs Conjugates
S10. MALDI-TOF Mass Spectrum for Molecular Characterization
Reference

## S1. Chemicals & Materials

The polymeric resins for library/peptide synthesis were acquired through commercial sources. TentaGel S $NH_2$ resin (90μm diameter, 0.31mmol/g $NH_2$ loading) was purchased from Rapp Polymere (Tubingen, Germany). Rinker amide MBHA resin (0.51mmol/g $NH_2$ loading) was purchased from P3 Biosystems (Louisville, KY). All resins were stored at 4°C before use.

All Fmoc-protected and Boc-protected amino acids in library/peptide synthesis were purchased through commercial sources. The vendors of Fmoc-protected amino acids include Aapptec Inc. (Louisville, KY), Chem-Impex International, Inc. (Wood Dale, IL), and P3 Biosystems (Louisville, KY). All amino acids were stored at 4°C and used as received without purification.

6-Chloro-1-hydroxy benzotriazole (6-Cl HOBt), 1-[Bis(dimethylamino)methylene]-1H-1,2,3-triazolo[4,5-b] pyridinium 3-oxide hexafluorophosphate (HATU) were purchased from Aapptec Inc. (Louisville, KY) and used as received without purification. N, N′-Diisopropylcarbodiimide (DIC), N, N-Diisopropylethylamine (DIEA), trifluoroacetic acid (TFA), Triisopropylsilane (TIS), myristic acid and acetic anhydride were purchased from Sigma Aldrich (St. Louis, MO). Phenylsilane was purchased from TCI America Inc (Portland, OR). Fluorescein isothiocyanate isomer I was purchased from Chem-Impex International, Inc. (WoodDale, IL) and stored at -20ºC in the dark before use.

NuPAGE™ 4 to 12% Bis-Tris precast mini-gel, Streptavidin-HRP conjugates, and Streptavidin-Alexa647 conjugated were purchased from ThermoFisher Scientific (Waltham, MA). Anti-HSA antibody alkaline phosphatase conjugate was purchased from Abcam (Cambridge, United Kingdom). Nitrocellulose membrane for western blot transfer, protein standard was purchased from Bio-Rad Laboratories (Hercules, CA). 5-Bromo-4-chloro-3-indolyl phosphate (BCIP) substrate, Human serum albumin (HSA), human serum, bovine serum albumin (BSA), and polysorbate 20 (Tween 20) were purchased from Sigma Aldrich (St. Louis, MO).

All proteins purchased were stored at optimal temperature based on afflicted specifications. Protein gels were used within the recommended shelf life.

# S2. Library Design & Synthesis

## S2.1. Library Design

The library was composed of 35 natural and unnatural amino acids summarized in Table S1

Table S1. Amino Acid Used in Library

| | | | |
|---|---|---|---|
| Fmoc- Aib-OH 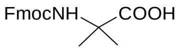 | Fmoc-Acpc-OH 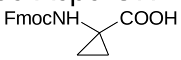 | Fmoc-L-HoCit 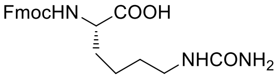 | Fmoc-Hyp(tBu)-OH 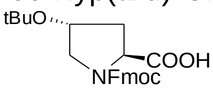 |
| Fmoc-Aad(OtBu)-OH 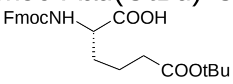 | Fmoc-D-3-Pal-OH 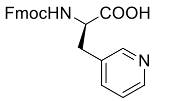 | Fmpc-Cha-OH 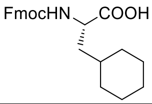 | Fmoc- Nva-OH 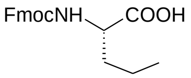 |
| Fmoc-Phe(3,4-diCl)-OH 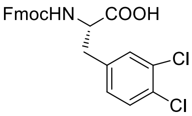 | Fmoc-D-Tyr(Me)-OH 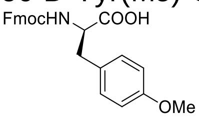 | Fmoc-L-Phg-OH 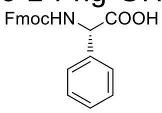 | Fmoc-Nle-OH 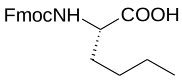 |
| Fmoc-L-Nal-1-OH 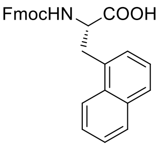 | Fmoc-Phe(4-Me)-OH 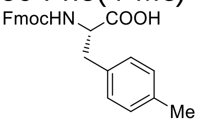 | Fmoc-Aic-OH 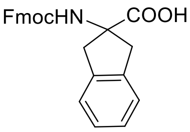 | Fmoc-D-Phe(3-Cl)-OH 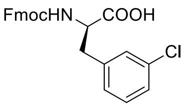 |
| Fmoc-D-HoPhe-OH 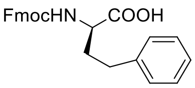 | Fmoc-D-Chg-OH 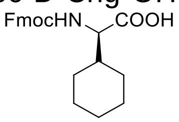 | Fmoc-Bpa-OH 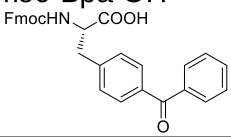 | Fmoc-D-Nal-2-OH 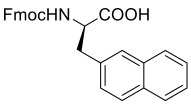 |
| Fmoc-Gly-OH 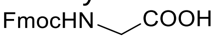 | Fmoc-D-Ser(tBu)-OH 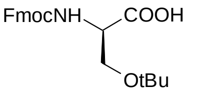 | Fmoc-Gln(Trt)-OH 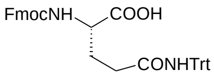 | Fmoc-D-Ala-OH 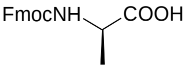 |
| Fmoc-D-Glu(OtBu)-OH 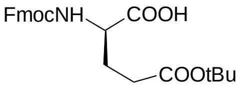 | Fmoc-D-Asn(Trt)-OH 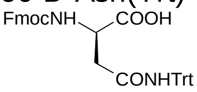 | Fmoc-Asp(OtBu)-OH 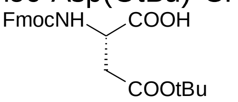 | Fmoc-Ile-OH 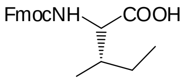 |
| Fmoc-D-Leu-OH 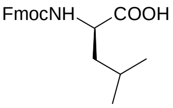 | Fmoc-Tyr(tBu)-OH 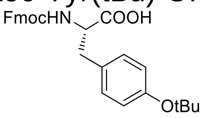 | Fmoc-D-Trp(Boc)-OH 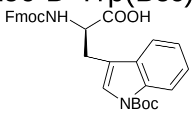 | Fmoc-D-Phe-OH 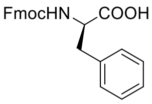 |
| Fmoc-D-Met-OH 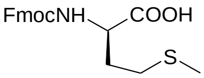 | Fmoc-D-Pro-OH 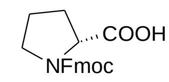 | Fmoc-L-Thr(tBu)-OH 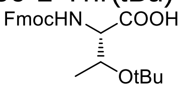 | Fmoc-L-Val-OH 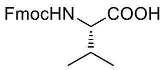 |

### S2.2 Library Synthesis

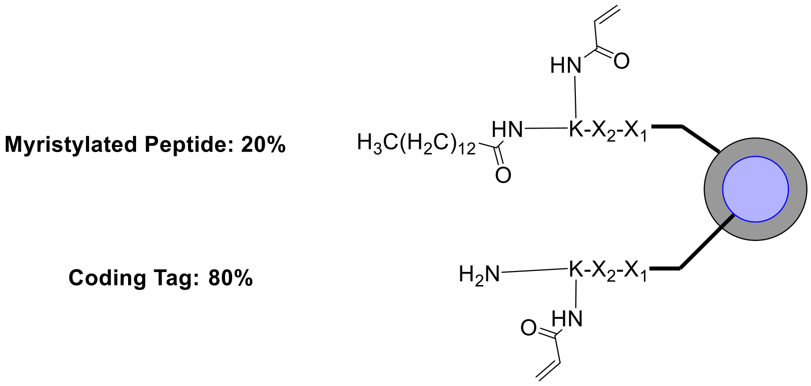


Figure S1 Tri-peptide $KX_2X_1$ based peptidomimetic OBOC Library

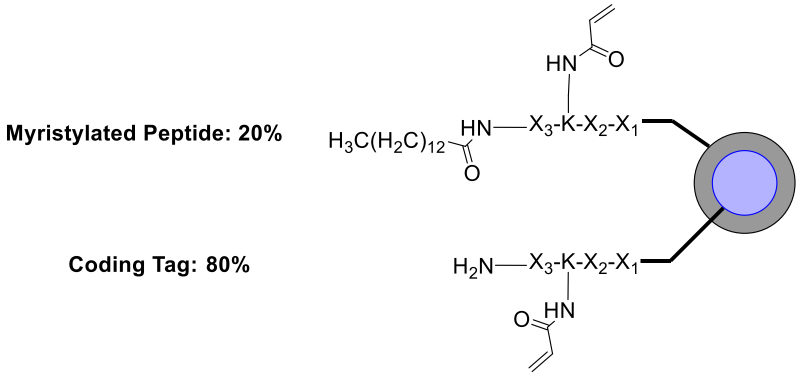


Figure S2 Tetra-peptide $X_3$ $KX_2X_1$ based peptidomimetic OBOC Library

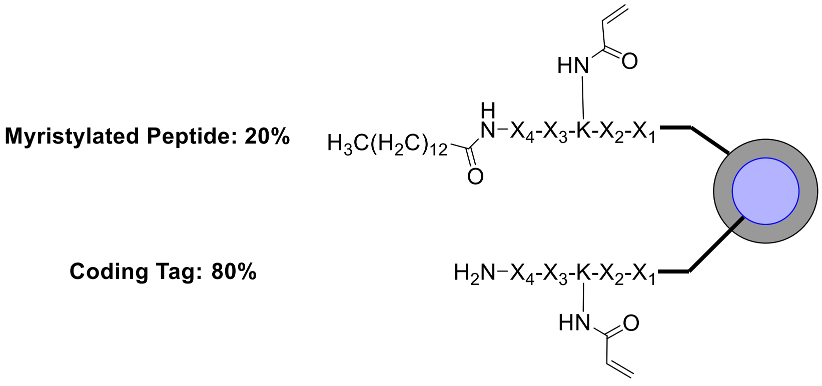


Figure S3 Penta-peptide $X_4X_3$ $KX_2X_1$ based peptidomimetic OBOC Library

Split-mix strategy was used in library synthesis to yield tri-, tetra- and pentapeptide based peptidomimetic libraries shown in Figures S1-S3, respectively. 2.0 g of TentaGel beads ($NH_2$ loading: 0.31 mmol/g) was swollen in DMF overnight before library synthesis. In brief, a library synthesis cycle starts with splitting the beads into 42 5mL plastic tubes equally. To each tube was added 1 mL of 0.2 M corresponding amino acid

solution, 1 mL of 0.2 M 6-Cl HOBt solution, and 50 μL of DIC. The coupling took 2 hours, and the completion of coupling was monitored by the ninhydrin test. After the coupling was done, the beads were combined in a 10 mL disposable polypropylene column with a polyethylene frit and the beads were washed with DMF, methanol and DMF, three times for each, before the addition of 20% 4-methyl piperidine in DMF to remove Fmoc protecting group (5 min, 15 min) and expose the N-terminal amine before next cycle began.

Before coupling myristic acid to the N-terminal of the peptides on beads, bi-layer beads were prepared using the bi-phasic solvent approach[1] to achieve 20% binding peptides displayed on the bead outer layer and 80% coding tag reside inside the beads. In brief, the library beads were dried completely and then swollen in water overnight. After water was drained, 178 mg Fmoc-OSu in 40 mL dichloromethane (DCM)/diethyl ether solution (v/v 55:45) was added to the beads, followed by addition of 86 μL DIEA. The column was vigorously shaken for 1 hour. The solution was drained, and the library beads were washed extensively by DMF and DCM. 432 mg Boc-anhydride in DCM was added to protect the *N*-terminal amino groups of coding peptides. After the Boc protection was done and confirmed by ninhydrin test, 20% 4-methyl piperidine in DMF was added to remove the Fmoc group, followed by coupling with myristic acid using 6-Cl HOBt/DIC. The Dde protecting group on lysine side chain in the library was then removed by 3% hydrazine monohydrate solution in DMF (5 min, 10 min). After extensive washing, 105 mg of acrylic acid (5 equiv.), 262 mg of 6-Cl HOBt (5 equiv.) and 239 μL of DIC (5 equiv.) was added to the beads. The coupling reached the completion in 1 hour.

The library beads were washed by DMF, MeOH, (DCM), three times each and dried under vacuum. Global deprotection was achieved with TFA cocktail (v/v, TFA: thioanisole: water: triisopropylsilane = 87.5%: 5% : 5% : 2.5%) for 3 hours. Then the TFA cocktail was drained, and the library beads were washed sequentially with DMF, DCM, MeOH, DMF, 50% DMF, water, ethanol, three time each, and stored in 70% ethanol for future screening.

## S3. Enzyme-linked Immunocolorimetric Screening Procedures

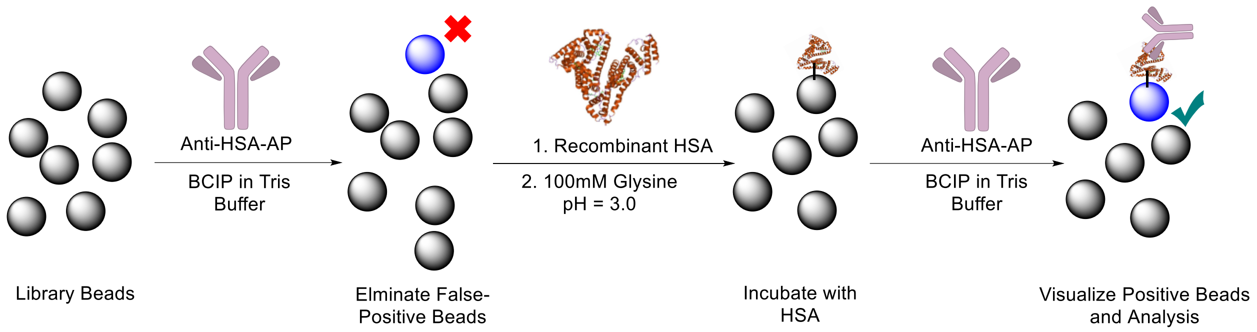


Figure S4 Schematic Illustration for Enzymatic Approach to Find RAE that Can Crosslink HSA

The screening for peptides that covalently target specific domain of HSA is performed in double-incubation approach: incubation with antibody solution to remove the false positive beads before incubation with HSA.

In a separatory column, approximately 100mg OBOC library (~300,000 beads) was washed by water for 3 times, followed by PBS-Tween 20-Gelatin buffer (PBSTG, made by 0.05% Tween 20 (v/v), 1% gelatin (w/v), pH = 7.4) for 3 times. The library was blocked by PBSTG for 1 hour at room temperature. Then the library was incubated with 2 mL 0.5 μg/mL Anti-HSA-Alkaline Phosphatase conjugate (Anti-HSA-AP) in PBSTG buffer for another 1 hour at room temperature. The antibody solution was drained, and the OBOC library beads were washed by TBS buffer twice. 1.65 mg BCIP substrate was dissolved in 10mL TBS buffer (pH = 8.8), and 1 mL of resulting substrate buffer was added to OBOC library beads. After 1 hour, beads with blue color were picked and discarded, and the rest beads were washed by 8 M guanidine HCl for 3 times, followed by washing with alternating DMF and methanol. Finally, the library beads were washed by PBSTG for 3 times and blocked by PBSTG buffer for another 1 hour. After that the library beads received 1 mL of 7.5 nM HSA solution in PBSTG buffer and the incubation last for 1 hour. Then the beads were washed by PBS for 3 times, followed by 3 times of 100 mM glycine solution (pH = 3.0) to elute non-covalently bound HSA. The antibody incubation and color development process were repeated as described above. Beads that bear blue color were picked and sequenced by Edman-degradation micro-sequencer.

# S4. Solid Phase Synthesis for RAEs and Derivatives

## S4.1. Synthesis of Biotinylated Reactive Affinity Elements

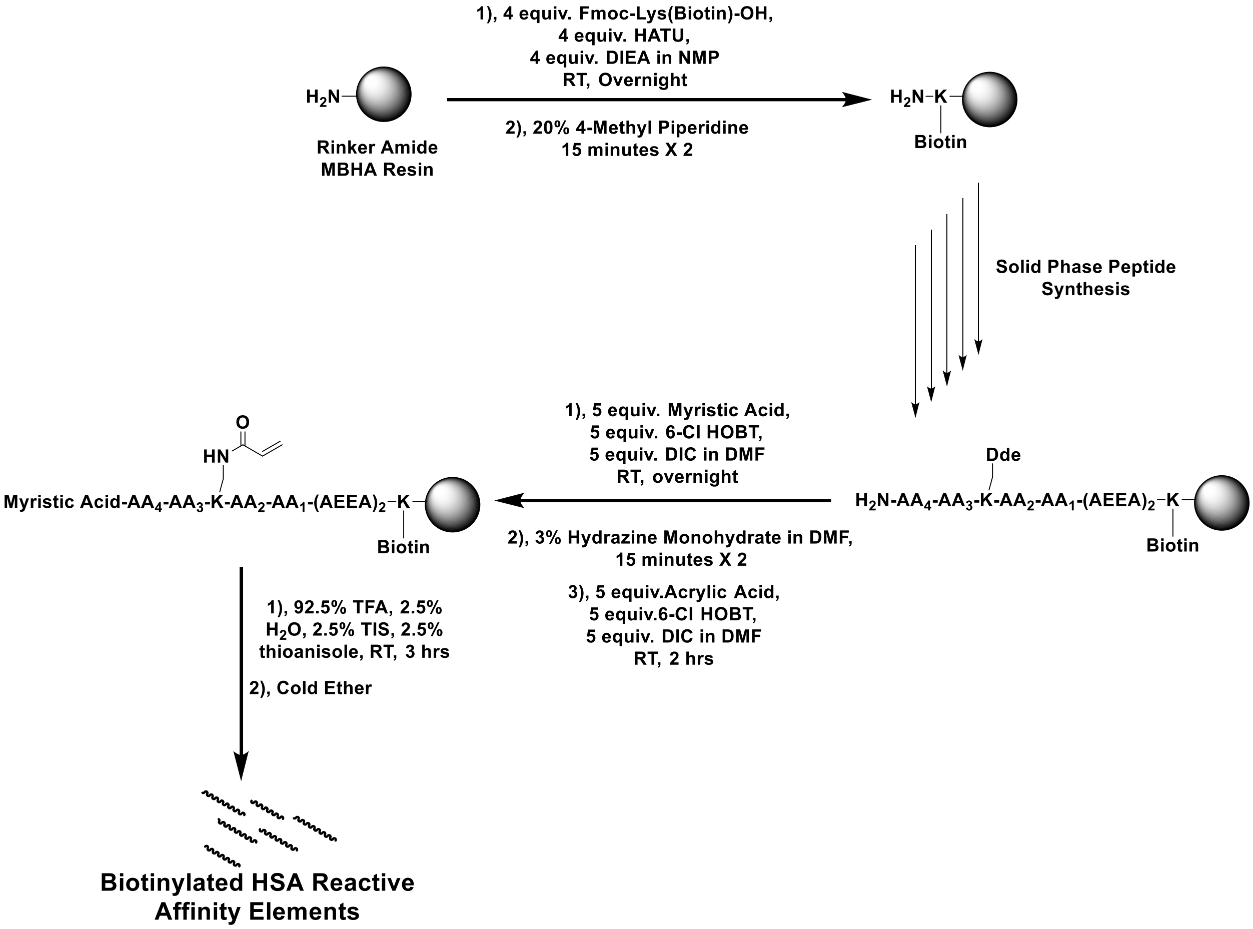


Scheme S1 Synthetic flow-chart for Biotinylated HSA Reactive Affinity Elements

Unless specified otherwise, the backbone of myristylated peptidomimetics was synthesized by microwave automatic peptide synthesizer (CEM) on Rinker amide MBHA resin ($NH_2$ loading: 0.51mmol/g) using 6 equivalents DIC/Oxyma and 6 equivalents of Fmoc-protected amino acids. Each coupling cycle took 2 minutes.

To prepare biotinylated reactive affinity elements and biotinylated non-covalent peptides used in binding affinity study, Fmoc-Lys (Biotin) was coupled to Rinker amide MBHA resin through 4 equivalents of HATU, Fmoc-Lys (Biotin)-COOH and DIEA in NMP solution at room temperature for overnight. The completion of coupling was monitored by ninhydrin test. After constructing the backbone of affinity elements using a peptide synthesizer (CEM Liberty Blue 2.0), myristoyl tail was installed on N-terminal of affinity

elements by 5 equivalents of myristic acid, 6-Cl HOBt and DIC for overnight at room temperature. To couple acrylic acid to lysine side chain, after the removal of Dde protecting group by 3% Hydrazine monohydrate, 5 equivalents of acrylic acid, 6-Cl HOBt and DIC were pre-mixed for half an hour before added to the resin. The coupling lasts for 1 hour at room temperature and the completion of coupling was monitored by ninhydrin tests.

To cleave the peptides off the beads, prior to cleavage the beads were washed with DMF, MeOH and DCM and thoroughly dried on vacuum. TFA cocktail (v/v, 95% TFA, 2.5% Water, 2.5% TIS) was prepared and added immediately to the dried beads and the mixture was shaken at a shaking bed for 2 hours. The TFA cocktail solution was then collected and condensed under nitrogen gas. Cold diethyl ether was added to precipitate the peptides and the resulting emulsion was enriched by centrifugation (3000 x g, 5 minutes). The ether was then discarded, and the peptides sediments were dissolved by acetonitrile/water mixture (50/50, v/v), followed by purification with Shimadzu LC-20AR Prominence liquid chromatograph suite coupled with a C18 column, starting at the mobile phase composed by ACN/Water = 70:30 containing 0.1% (v/v) TFA. The fractions containing desired products were collected or combined. The solvent was removed by lyophilization.

**S4.2. Synthesis of FC-LYL1**

The FC-LYL1 was synthesized from intermediate LYL1-K($NH_2$) that is prepared by similar solid phase peptide synthesis (SPPS) strategy described in S4.1. The LYL1-K($NH_2$) intermediate was cleaved off the resin by TFA cocktail and purified by reverse-phase HPLC. The purified LYL1-K($NH_2$) was crosslinked to FITC through amino-thiocyanate addition in the presence of 2 equivalents of DIEA (Scheme S2).

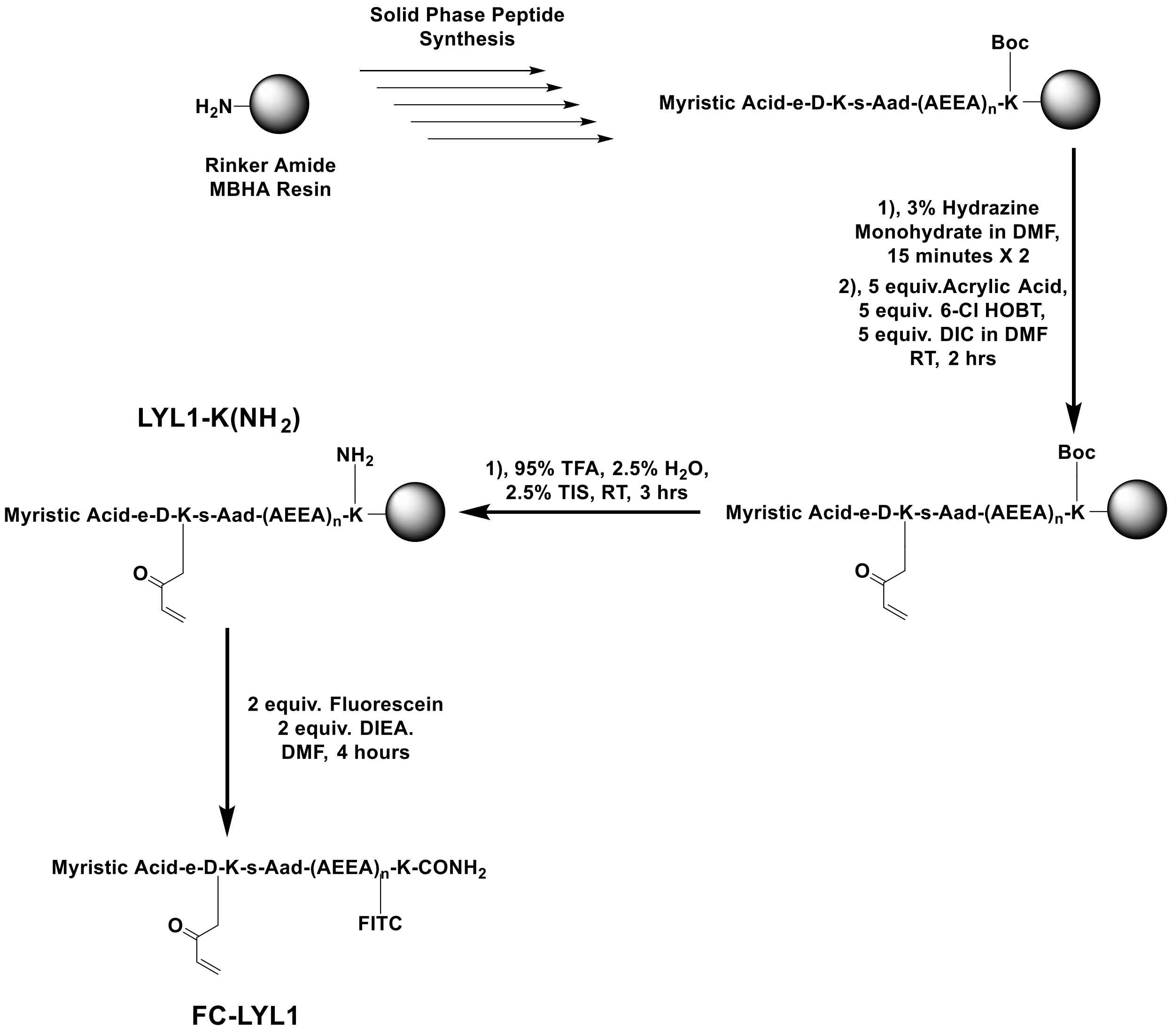


Scheme S2 Synthesis of FC-LYL-1

## S4.3. Synthesis of LLP2A-$(EBES)_6$-Mal

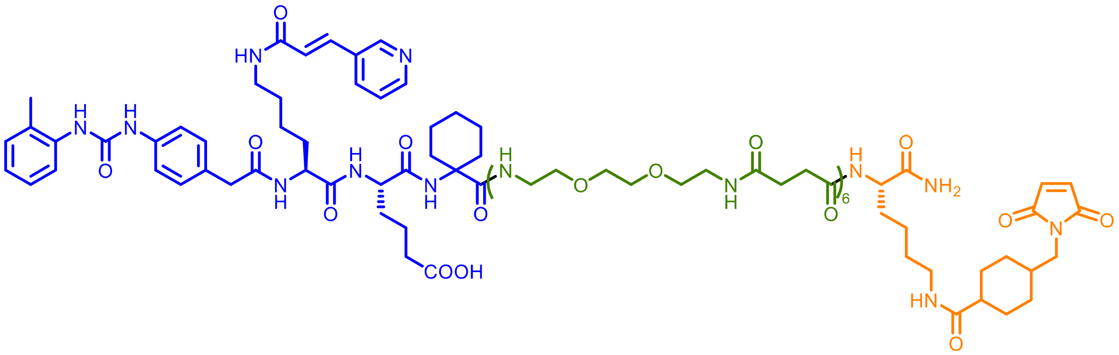


Figure S5 Chemical Structure of LLP2A-$(EBES)_6$-Mal

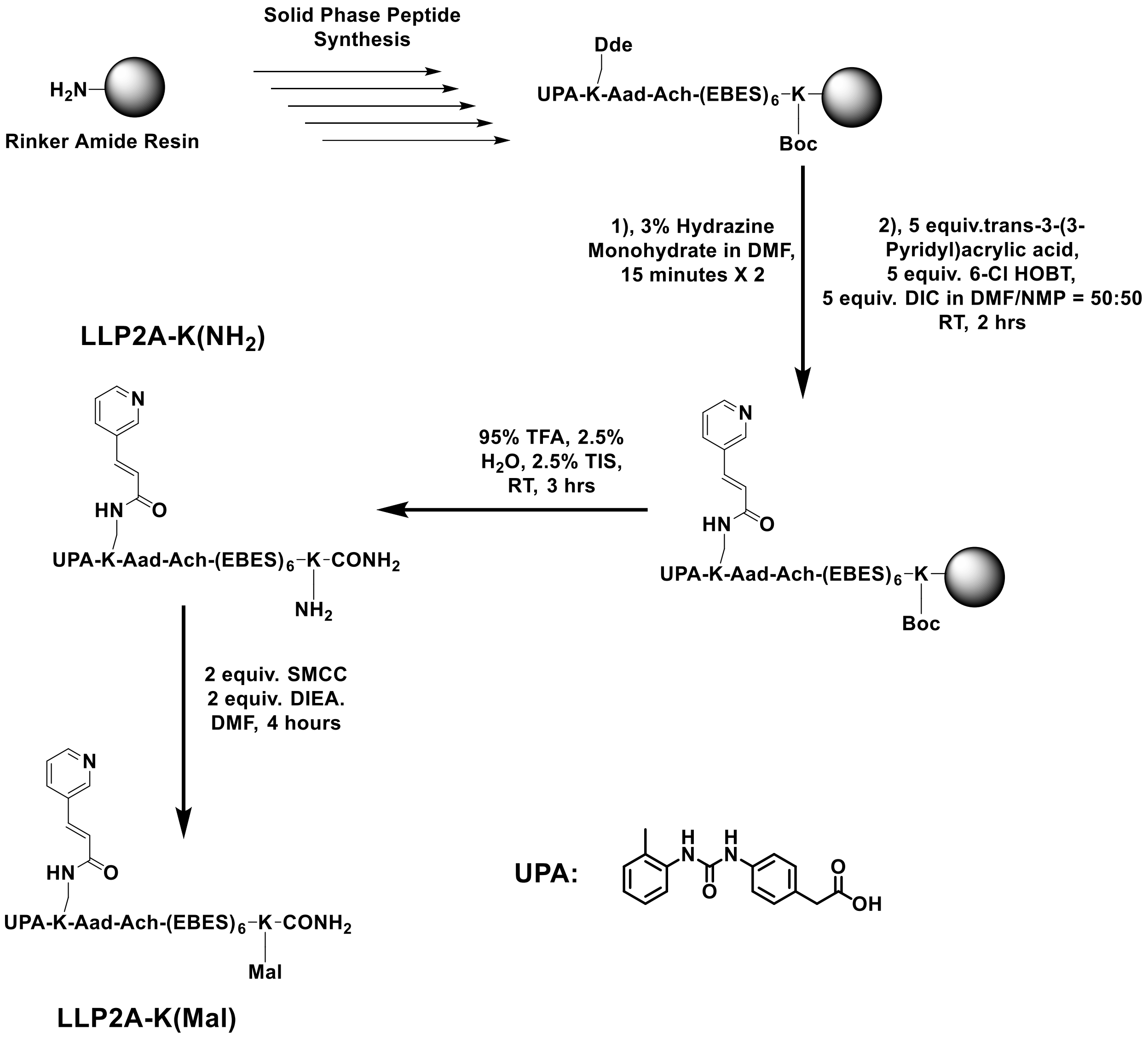


Scheme S3 Synthesis of LLP2A-Mal

The LLP2A-(EBES)6-Mal was prepared from the intermediate (LLP2A-(EBES)6-K($NH_2$)) that is synthesized by SPPS strategy as shown in Scheme S3. After purification of intermediate LLP2A-K($NH_2$), maleimide was coupled to the amino group of lysine through 2 equivalents of succinimidyl 4-(N-maleimidomethyl) cyclohexane-1-carboxylate (SMCC) in the presence of 2 equivalents of DIEA. The chemical structure is shown in Figure S5.

## S5. BLI Assay for Biotinylated Non-covalent Peptidomimetics.

**NCB-LYL1**

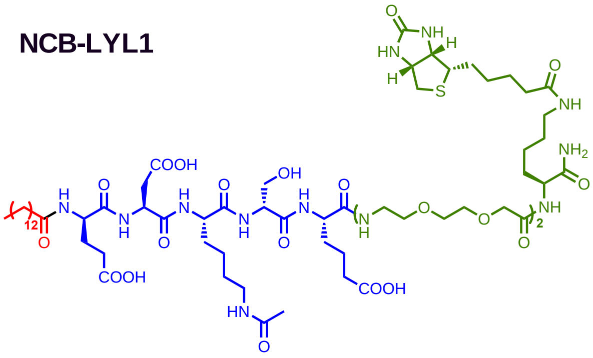

**NCB-LYL2**

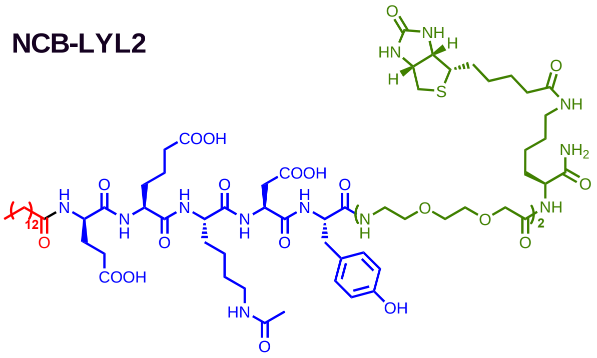

**NCB-LYL3**

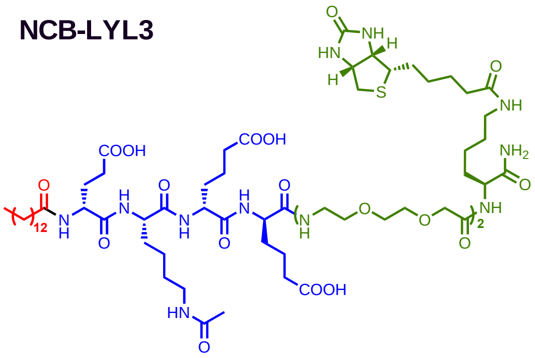

**NCB-LYL4**

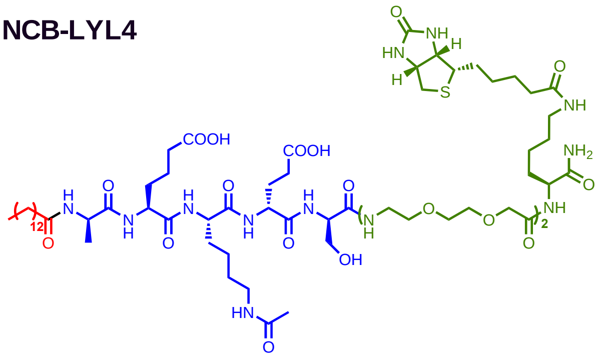

**NCB-LYLG**

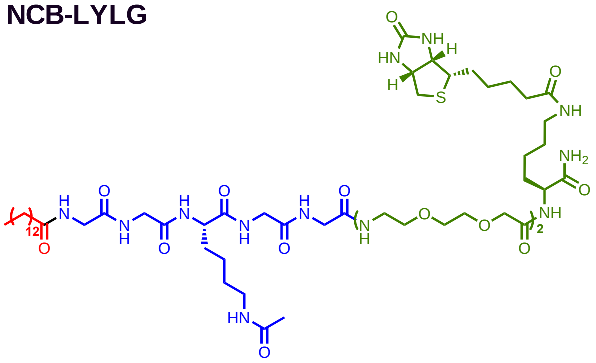

Figure S6 Chemical Structure of Non-covalent Biotinylated RAEs (NCB-LYL1-4, NCB-LYLG)

Biotinylated Non-covalent RAEs was synthesized by replacing acrylamide with acetyl group to study the intrinsic binding affinity of peptidomimetics using bio-layer interferometry (BLI) assay. For BLI assay, 1 μM non-covalent biotinylated RAEs (Figure S6) solution was prepared by kinetic buffer as the bait. As the fish, HSA solution at various concentration prepared by serial dilution using kinetic buffer, and the last well of dilution was filled with kinetic buffer as the reference well. Using 96-well plate, the biosensors were equilibrated in kinetic buffer for 60s. Then biosensors dipped ligands

wells, kinetic buffer wells, HSA well, kinetic buffer wells again for 120s, 60s, 480s, 960s as loading, baseline, association, and dissociation step, respectively. The data was subtracted by reference wells and processed by ForteBio Data Analysis software to derive the Kd value using steady-state approximation by 1:1 model (Figure S7-S8)

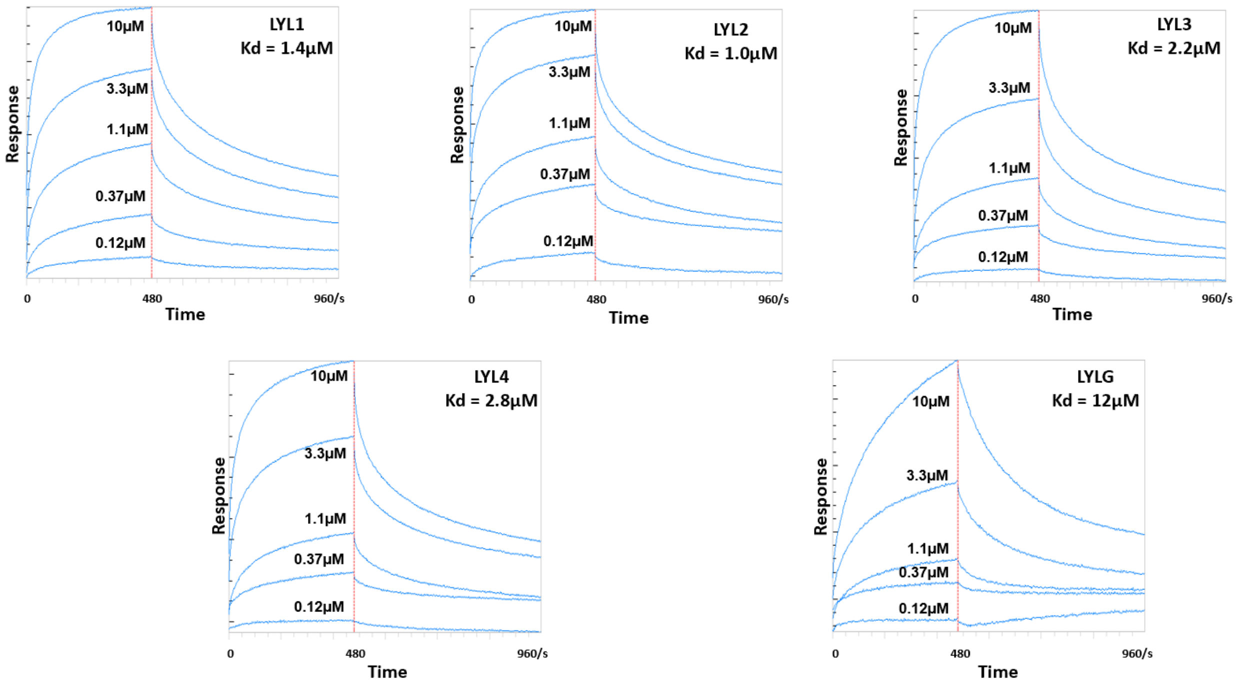


Figure S7 Bio-layered Interferometry for NCB-LYL1-4 and NCB-LYL-G

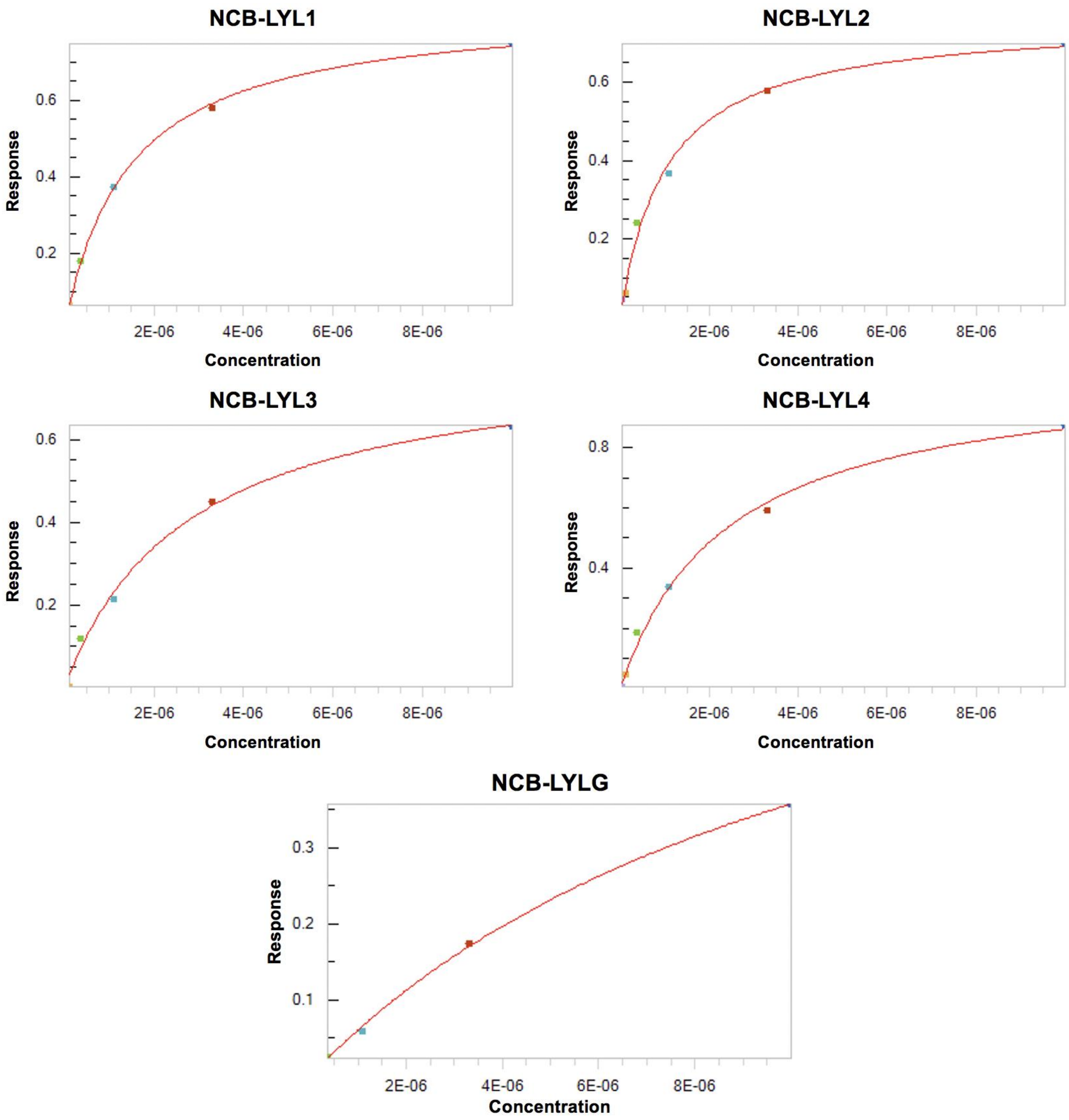


Figure S8 Stead State Approximation Fitting for Binding between Non-covalent RAE with HSA

## S6 Quantification of Biotinylated HSA using Neutravidin Pull-Down Assay

### S6.1 General Procedures to Purify Biotinylated Human Serum Albumin

Zeba spin desalting columns were pre-equilibrated by PBS buffer 3 times on centrifugation (1500 x g, 1 minute). After conjugation, HSA-LYL conjugates passed through the equilibrated Zeba desalting column. The flow through was collected and further condensed by a 500 μL ultracentrifugation tube (MW cutoff = 3000) at 15000 rpm at 4°C for 15 minutes. The resulting condensed solution was immediately reconstituted by PBS (pH = 7.2), and the condensation process was repeated 2 times to further remove biotinylated peptidomimetics. After that, the condensed HSA conjugate (typically 20-50 μL) was diluted by 100 μL PBS for Neutravidin pull-down assay.

### S6.2. General Procedure to Determine Conjugation Yield by Neutravidin Pull-Down Assay

Using neutravidin-immobilized agarose, the NeutrAvidin pull-down assay intercepted biotinylated proteins through biotin-avidin interaction. Therefore, the conjugation yield can be determined by measuring the difference caused by NeutrAvidin pull-down.

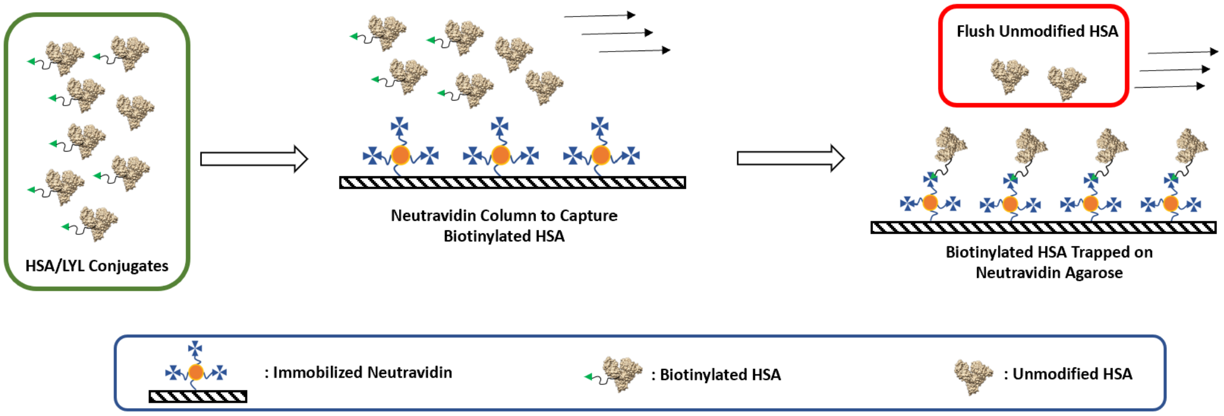


Scheme S4 Schematic Demonstration for Neutravidin Pull-Assay

The yield of conjugation is calculated based on the concentration difference of HSA before and after passing through Neutrvidin agarose, which is calculated by following equation (1):

$$Yield = \frac{c_{HSA\ Stock} - c_{HSA\ Flow-through}}{c_{HSA\ Stock}} \times 100\% \qquad (1)$$

To perform the pull-down assay, 100 µL slurry of high capacity NeutrAvidin agarose (ThermoFisher Scientific) was loaded into a separatory tube. The resin was equilibrated by binding buffer (PBS buffer, 0.1 M sodium phosphate, 0.15 M sodium chloride, pH = 7.2) and washed under centrifugation at low speed (500 x g) for 1 minute. The washing was repeated 3 times. The agarose resin was then dried completely at high speed (5000 x g) for 2 minutes to remove water residue. After that, 100 µL purified HSA-LYL conjugate was added, and the resulting mixture was incubated at room temperature for 30 minutes. The flow-through solution was collected by centrifugation and the HSA concentration was determined by Bicinchoninic acid (BCA) assay. 3 replications were performed for pull-down assay for error analysis.

### S6.3. Bicinchoninic acid (BCA) Assay

BCA assay was performed using Pierce™ BCA Protein Assay kit to determine the protein concentration in matrix. The BCA working solution was used immediately after preparation. HSA solutions at the concentration of 25.00 µM, 12.50 µM, 6.25 µM, 3.13 µM, 1.56 µM and 0 µM were prepared by binding buffer for calibration curve. The coulometric reactions were performed at 68°C for 30 minutes and the UV absorbance at 562 nm was measured by plate reader.

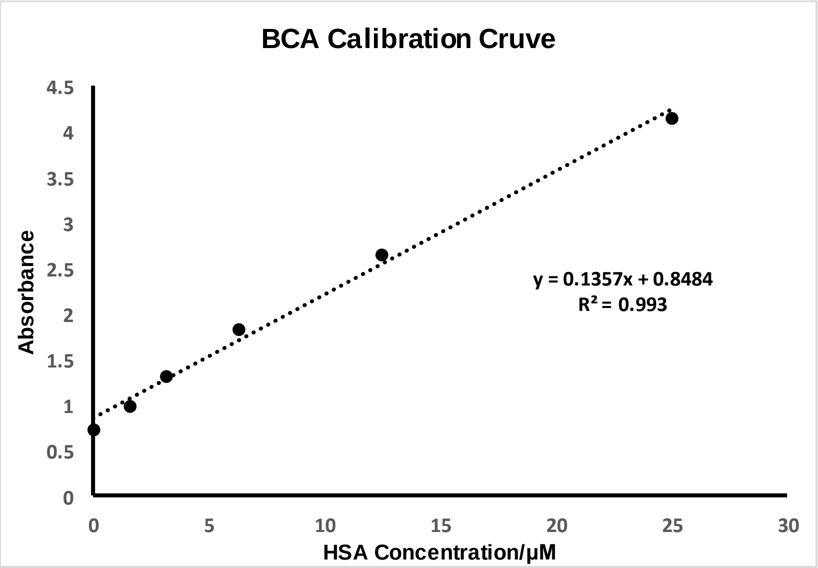


Figure S9 BCA Assay Calibration Curve

### S6.4. Validation of NeutrAvidin Pull-Down Assay

To 200 µL PBS buffer (0.1 M sodium phosphate, 0.15 M sodium chloride, pH = 7.2) was added 1 µL HSA stock solution and 1µL RAE or 1 µL NBE stock solution to prepare conjugation solution containing 10 µM HSA and 50 µM RAE or 50 µM NBE. The solution was incubated at room temperature for 1 hour. Blank solution was prepared by 10 µM native HSA without modification. The conjugation yield has good relationship with fluorescence intensity of corresponding Western blots. As NeutrAvidin demonstrated very low non-specific binding towards native HSA (< 2% uptake), the conjugation yield was calculated without background subtraction.

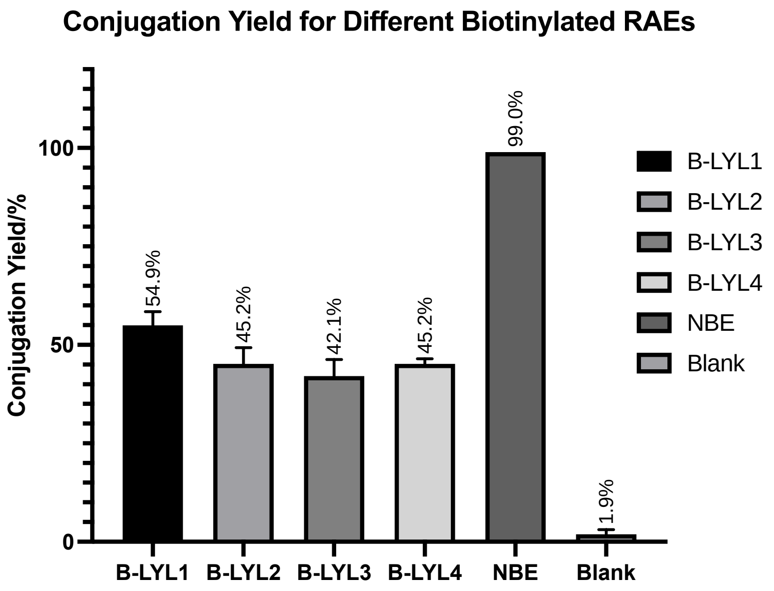


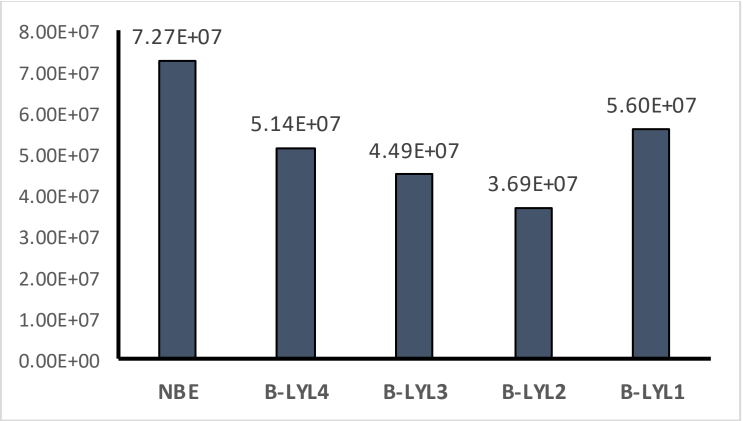


Figure S10 NeutrAvidin Pull-Down Assay Results for Different Biotinylated RAEs (Up) and Western Blots Intensity (Bottom) for Different RAEs, NBE and Blank

## S6.5. Conjugation Yield at Different pH

To 200 µL PBS buffer (0.1 M sodium phosphate, 0.15 M sodium chloride), at different pH was added 1 µL HSA stock solution and 1 µL RAE stock solution to prepare conjugation solution containing 10 µM HSA and 50µM RAE. The solution was incubated at room temperature for 1 hour.

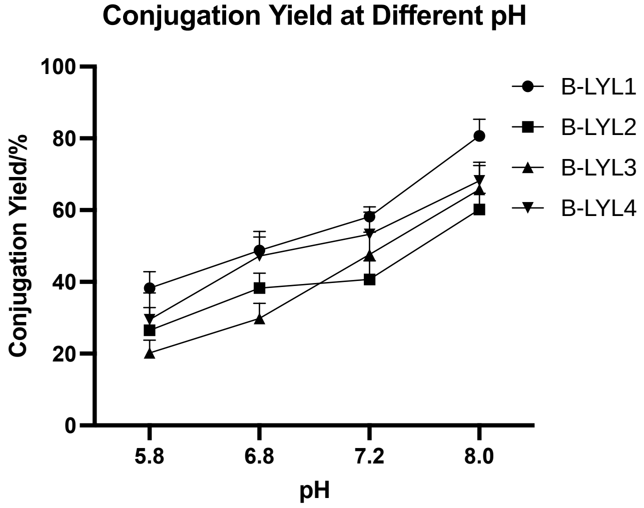


Figure S11 NeutrAvidin Pull-down Assay Showed Conjugation Yield for B-LYL1-4 is pH Dependent. Alkaline pH Promotes Degree of Conjugation

| | | Biotinylated RAEs Conjugation Yield/% | | | |
|---|---|---|---|---|---|
| | | **B-LYL1** | **B-LYL2** | **B-LYL3** | **B-LYL4** |
| **pH** | **5.8** | 38.2 | 26.5 | 20.2 | 30.0 |
| | **6.8** | 48.7 | 38.3 | 29.8 | 47.2 |
| | **7.2** | 58.2 | 40.7 | 47.6 | 53.3 |
| | **8.0** | 81.6 | 60.2 | 65.7 | 68.2 |

Table S2 Conjugation Yield for B-LYL1-4 at Different pH

## S6.6. Conjugation Yield at Various Concentration

To PBS buffer (0.1 M sodium phosphate, 0.15 M sodium chloride) at pH = 7.2 or pH = 8.0 was added 1 µL HSA stock solution and various amounts of RAE stock solution to prepare conjugation solution containing 10 µM HSA and 5 µM, 10 µM, 20 µM, 50 µM, 100 µM, 150 µM and 200 µM RAEs. Additional PBS buffer was added to make the overall volume of the solution to be 200 µL. The solution was incubated at room temperature for 1 hour.

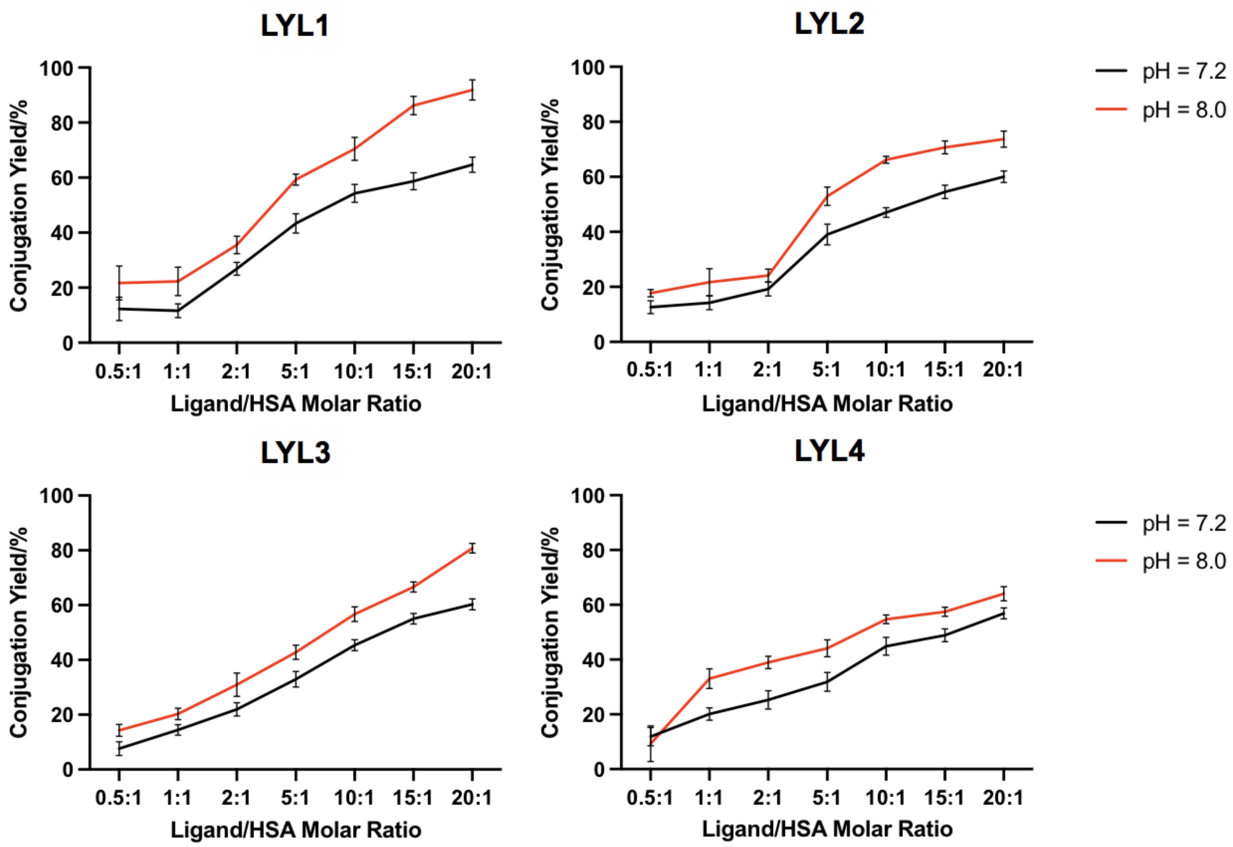


Figure S12 NeutrAvidin Pull-down Assay Showed Conjugation Yield for B-LYL1-4 is Higher at Elevated RAEs Concentration

| | | B-LYL1 Concentration/μM | | | | | | |
|---|---|---|---|---|---|---|---|---|
| | | 5 | 10 | 20 | 50 | 100 | 150 | 200 |
| pH | 7.2 | 12.3 | 11.7 | 26.9 | 43.4 | 54.3 | 58.7 | 64.7 |
| | 8.0 | 21.7 | 22.3 | 35.5 | 59.2 | 70.4 | 86.2 | 91.9 |

| | | B-LYL2 Concentration/μM | | | | | | |
|---|---|---|---|---|---|---|---|---|
| | | 5 | 10 | 20 | 50 | 100 | 150 | 200 |
| pH | 7.2 | 12.6 | 14.2 | 19.1 | 39.0 | 47.0 | 54.5 | 60.0 |
| | 8.0 | 17.7 | 21.7 | 24.1 | 52.9 | 66.2 | 70.7 | 73.7 |

| | | B-LYL3 Concentration/μM | | | | | | |
|---|---|---|---|---|---|---|---|---|
| | | 5 | 10 | 20 | 50 | 100 | 150 | 200 |
| pH | 7.2 | 7.6 | 14.4 | 22.0 | 33.0 | 45.4 | 55.1 | 60.3 |
| | 8.0 | 14.3 | 20.3 | 31.0 | 42.8 | 56.7 | 66.6 | 80.8 |

| | | B-LYL4 Concentration/μM | | | | | | |
|---|---|---|---|---|---|---|---|---|
| | | 5 | 10 | 20 | 50 | 100 | 150 | 200 |
| pH | 7.2 | 11.9 | 20.1 | 25.3 | 31.9 | 44.9 | 48.9 | 56.9 |
| | 8.0 | 9.3 | 33.0 | 39.0 | 44.1 | 54.7 | 57.5 | 64.1 |

Table S3 Conjugation Yield at Different RAEs Concentration

### S6.7. Conjugation Yield at for Different Time

To 197 μL PBS buffer at pH = 7.2 or pH = 8.0 was added 1 μL HSA stock solution and 2 μL RAEs stock solution prepare conjugation solution containing 10 μM HSA and 100 μM RAEs. The solution was incubated at room temperature for 1 hour, 2 hours, 3 hours, 4 hours, 5 hours and 24 hours, respectively. The conjugation yield was determined by pull-down assay.

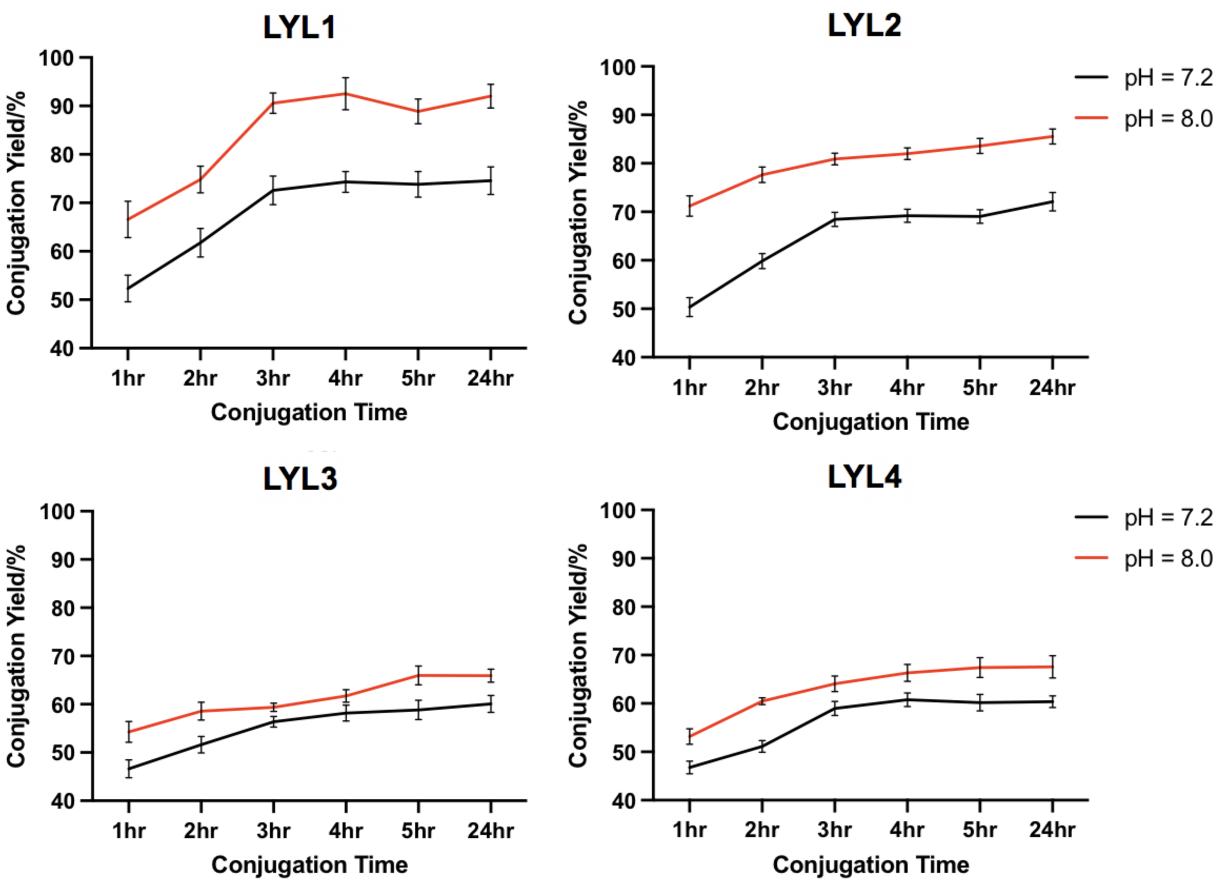


Figure S13 NeutrAvidin Pull-down Assay Showed Conjugation Yield for B-LYL1-4 is Higher at Prolonged Conjugation Time

| | | B-LYL1 Conjugation Time/hr | | | | | |
|---|---|---|---|---|---|---|---|
| | | **1** | **2** | **3** | **4** | **5** | **24** |
| **pH** | **7.2** | 52.3 | 61.8 | 72.6 | 74.3 | 73.9 | 74.6 |
| | **8.0** | 66.6 | 74.8 | 90.6 | 92.5 | 88.9 | 92.0 |

| | | B-LYL2 Conjugation Time/hr | | | | | |
|---|---|---|---|---|---|---|---|
| | | **1** | **2** | **3** | **4** | **5** | **24** |
| **pH** | **7.2** | 50.3 | 59.9 | 68.4 | 69.2 | 69.0 | 72.0 |
| | **8.0** | 71.2 | 77.6 | 80.9 | 82.0 | 83.6 | 85.5 |

| | | B-LYL3 Conjugation Time/hr | | | | | |
|---|---|---|---|---|---|---|---|
| | | 1 | 2 | 3 | 4 | 5 | 24 |
| pH | 7.2 | 46.6 | 51.6 | 56.4 | 58.2 | 58.8 | 60.1 |
| | 8.0 | 54.3 | 58.6 | 59.3 | 61.7 | 66.0 | 65.9 |

| | | B-LYL4 Conjugation Time/hr | | | | | |
|---|---|---|---|---|---|---|---|
| | | 1 | 2 | 3 | 4 | 5 | 24 |
| pH | 7.2 | 46.8 | 51.1 | 59.0 | 60.8 | 60.2 | 60.4 |
| | 8.0 | 53.1 | 60.5 | 64.1 | 66.3 | 67.4 | 67.6 |

Table S4 Conjugation Yield at Different Conjugation Time

# S7. Tryptic Digestion and Proteomics Analysis

## S7.1 Tryptic Digestion

HSA-BLYL1 conjugate was prepared by 1mM HSA solution and 10 mM B-LYL1 solutions at alkaline pH (pH = 8.0) in room temperature with molar ratio HSA : Biotin-LYL1 = 1:10 for overnight. The conjugate was purified through desalting column for solvent exchange and condensed by ultracentrifugation before trypsin digestion.
The HSA conjugates were reconstituted in approximately 100μL of 6.0M urea solution. Then the reducing reagent (DTT stock solution) was added to a final concentration of 5mM DTT. The mixture was incubated at 37°C for 30 minutes before the addition of alkylating reagent (Iodoacetamide, IAM) to a final concentration of 15mM. The alkylation took place at room temperature in dark for 30 minutes, which was later quenched by DTT stock solution. The Lys-C/trypsin was added in a 1:25 (enzyme:protein) ratio and incubate at 37°C for 4 hours. After that 550 μL 50mM ammonia bicarbonate (AMBIC) solution was added to dilute the urea and activate trypsin digestion for overnight at 37°C. The conjugates were cleaned-up by MiniSpin™ columns (The Nest Group., Inc) for desating by LC-MS water and acetonitrile.

## S7.2 Database Searching Parameters

Charge state deconvolution and deisotoping were not performed. All MS/MS samples were analyzed using X! Tandem (The GPM, thegpm.org; version X! Tandem Alanine (2017.2.1.4)). X! Tandem was set up to search the 20190304-human-FRDB-wcrap-wP62 database (unknown version, 147956 entries) assuming the digestion enzyme trypsin. X! Tandem was searched with a fragment ion mass tolerance of 20 PPM and a parent ion tolerance of 20 PPM. Carbamidomethyl of cysteine and selenocysteine was specified in X! Tandem as a fixed modification. Glu->pyro-Glu of the n-terminus, ammonia-loss of the n-terminus, gln >pyro-Glu of the N-terminus, deamidated of asparagine and glutamine, oxidation of methionine and tryptophan, dioxidation of methionine and tryptophan, c+1528 of cysteine and k+1528 of lysine were specified in X! Tandem as variable modifications.

## S7.3. LC/MSMS results

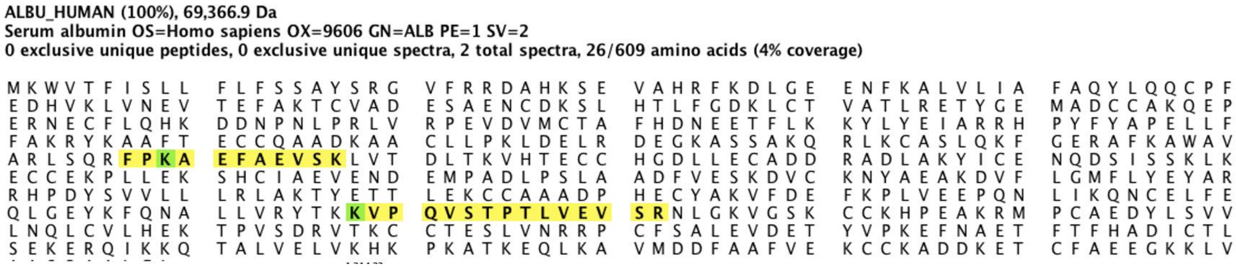


Figure S14 HSA Sequence

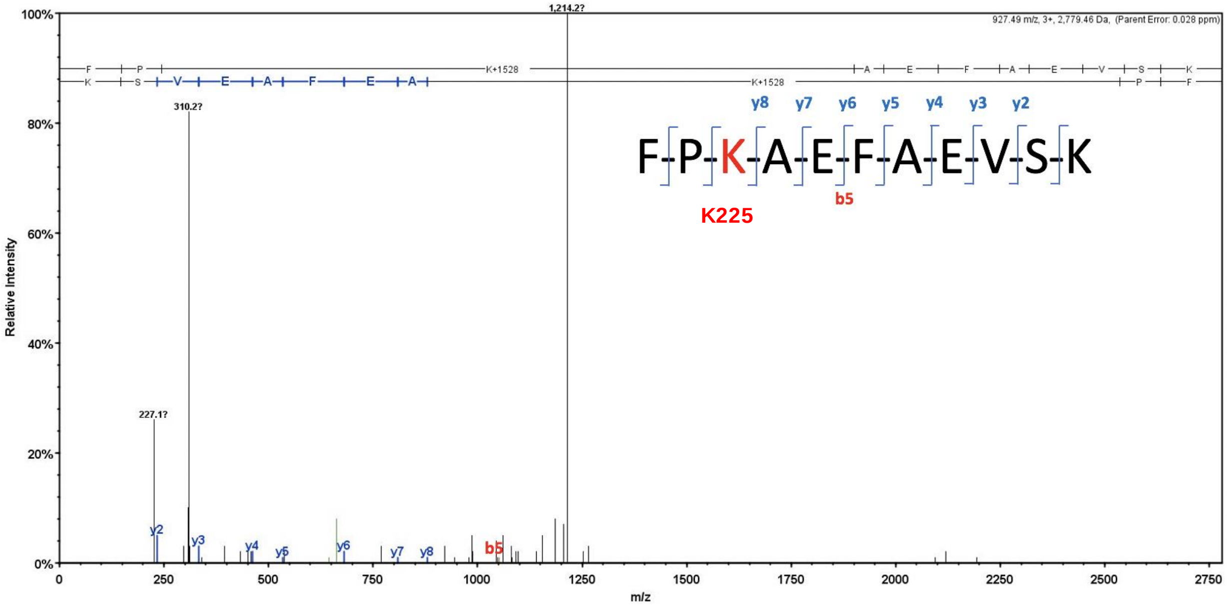


Figure S15 B-LYL1 Adduct at K225. Peptides at 927.49m/z (2779.47 + 3H$^+$) was fragmented

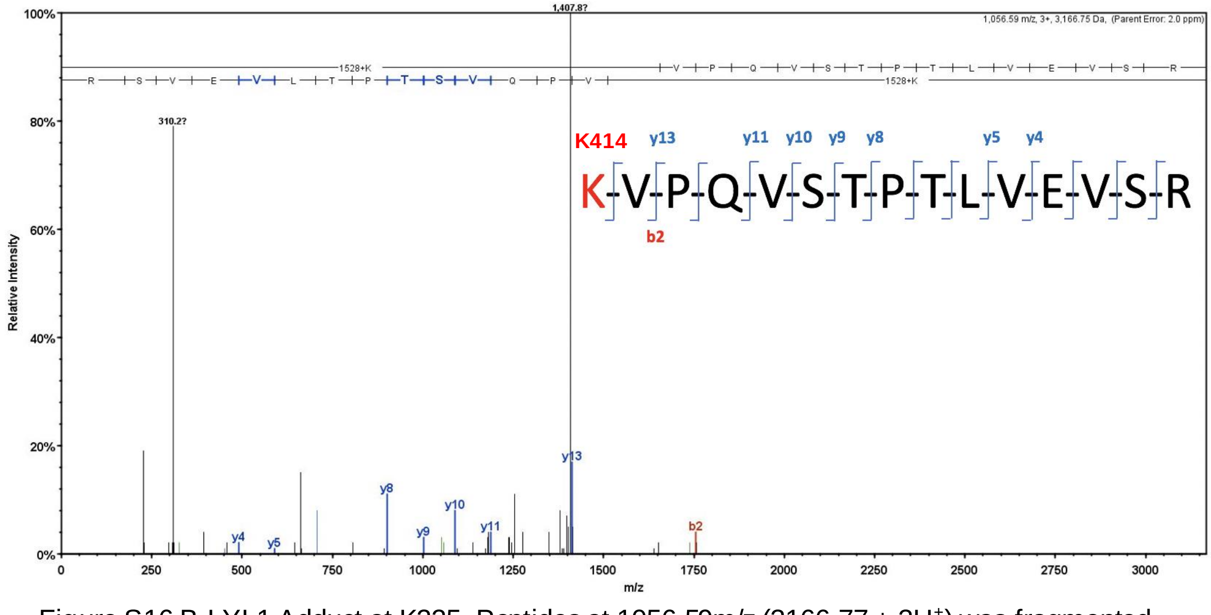


Figure S16 B-LYL1 Adduct at K225. Peptides at 1056.59m/z (3166.77 + 3H$^+$) was fragmented

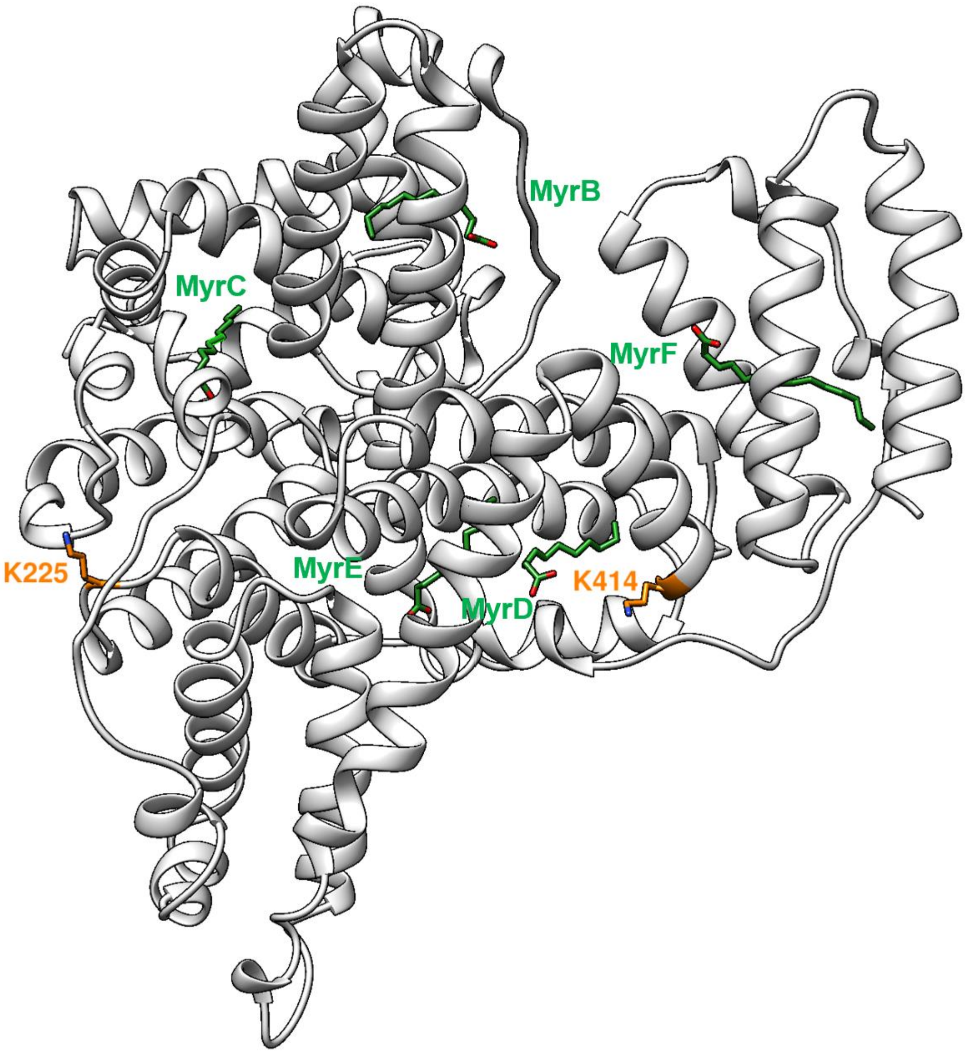


Figure S17 Location of K414 and K225 in HSA-Myristate complex (1BJ5). The sites of ligation are close to the myristate binding sites where myristate (MyrB-E) bind.

## S8 Computational Prediction for Protonation States of HSA

The pK (1/2) and protonation states of titratable amino acid residues of HSA was calculated on H++ severer based on 3 different crystal structures of native HSA (PDB ID: 1AO6)[3] and HSA-myristate complex (PDB ID: 1BJ5 and 4L8U).[4-5] The prediction was performed at pH = 7.2, with salinity of 0.15 M, internal dielectric constant at 10 and external dielectric constant at 80. The predicted pK int (hypothetical pK of a group assuming that it does not interact with any other titratable group) and pK (1/2) (mid-point of a titration curve) was summarized in Table S7.

The calculated pK (1/2) values are compared with previous calculation[6] and experiments results.[7] K199 has lowest pKa value for 1BJ5 and 1AO6, while the pKa of K564 is lowest in 4L8U. The pKa values for K41, K73, K106, K233, K240 and K534 are higher than 12 for all 3 models.

Table S5 Predicted pK Int and pK (1/2) for HSA-Myristate Complex at neutral pH

| | 1BJ5 | | 1AO6* | | 4L8U | | Remark |
|---|---|---|---|---|---|---|---|
| | pK int | pK (1/2) | pK int | pK (1/2) | pK int | pK (1/2) | |
| LYS-12 | 9.92 | 11.19 | 10.08 | 11.91 | 9.97 | 11.08 | |
| LYS-20 | 9.72 | >12.00 | 9.70 | >12.00 | 9.74 | 11.95 | |
| LYS-41 | 10.10 | 11.20 | 9.82 | 11.43 | 10.22 | >12.00 | |
| LYS-51 | 10.06 | 11.74 | 9.66 | 11.12 | 9.67 | >12.00 | |
| LYS-64 | 10.00 | 11.67 | 10.23 | 11.58 | 10.42 | 11.95 | Calculation from Previous Literature: pKa = 11.070 |
| LYS-73 | 9.54 | >12.00 | 9.50 | >12.00 | 9.77 | >12.00 | |
| LYS-93 | 9.89 | 11.08 | 9.60 | 11.00 | 9.80 | 11.09 | |
| LYS-106 | 10.08 | >12.00 | 10.03 | >12.00 | 10.22 | >12.00 | |
| LYS-136 | 8.87 | 11.87 | 9.21 | 10.44 | 9.44 | 9.81 | |
| LYS-137 | 9.65 | 11.95 | 9.64 | 10.61 | 9.91 | 10.99 | |
| LYS-159 | 8.81 | 9.12 | 8.97 | 12.00 | 9.72 | 10.72 | |
| LYS-162 | 9.56 | 11.73 | 9.94 | 11.85 | 9.83 | 11.42 | |
| LYS-174 | 9.65 | 11.21 | 9.52 | 11.08 | 9.99 | 10.97 | |
| LYS-181 | 9.48 | 11.93 | 9.25 | 11.11 | 9.96 | 11.68 | |
| LYS-190 | 9.85 | 9.45 | 9.30 | 6.88 | 9.95 | 10.25 | |
| LYS-195 | 9.94 | >12.00 | 9.90 | 11.97 | 9.93 | >12.00 | |
| LYS-199 | 9.26 | 8.25 | 8.85 | 6.23 | 9.42 | 8.95 | Lowest pKa for 1BJ5 and 1AO6; Experimentally most reactive lysine for Michael |

| | | | | | | | |
|---|---|---|---|---|---|---|---|
| | | | | | | | Addition; |
| LYS-205 | 10.56 | 9.65 | 10.69 | 11.62 | 10.44 | 10.79 | |
| LYS-212 | 9.53 | 11.42 | 9.65 | 10.98 | 9.80 | 11.36 | |
| LYS-225 | 9.64 | >12.00 | 9.62 | 11.57 | 9.72 | >12.00 | Site I |
| LYS-233 | 9.30 | >12.00 | 9.82 | >12.0 | 9.80 | >12.00 | |
| LYS-240 | 10.08 | >12.00 | 9.90 | >12.00 | 9.88 | >12.00 | |
| LYS-262 | 10.15 | 11.94 | 10.04 | >12.00 | 10.19 | >12.00 | |
| LYS-274 | 9.67 | 11.43 | 9.43 | >12.00 | 10.06 | >12.00 | |
| LYS-276 | 10.22 | 11.09 | 9.94 | 11.26 | 10.24 | 11.77 | |
| LYS-281 | 9.79 | >12.00 | 9.95 | 11.10 | 9.93 | >12.00 | |
| LYS-286 | 9.85 | 11.24 | 9.93 | 11.22 | 9.55 | 10.92 | |
| LYS-313 | 9.98 | 11.07 | 10.18 | 11.02 | 10.20 | 11.23 | |
| LYS-317 | 10.05 | 10.90 | 10.11 | 11.48 | 10.23 | 11.12 | |
| LYS-323 | 10.10 | >12.00 | 9.98 | 11.75 | 10.08 | >12.00 | |
| LYS-351 | 9.71 | 10.96 | 9.48 | 11.12 | 9.52 | 9.89 | |
| LYS-359 | 10.38 | 10.96 | 10.18 | 10.83 | 10.34 | 11.11 | |
| LYS-372 | 10.02 | 10.95 | 10.11 | 11.11 | 10.22 | 11.30 | |
| LYS-378 | 10.12 | 11.43 | 10.09 | 11.25 | 10.17 | 11.96 | |
| LYS-389 | 10.23 | 11.16 | 10.11 | 10.82 | 10.18 | 11.64 | |
| LYS-402 | 10.03 | 10.27 | 10.23 | 10.96 | 10.20 | 10.52 | |
| LYS-413 | 10.49 | 10.60 | 10.53 | 10.48 | 11.55 | >12.00 | |
| LYS-414 | 10.06 | 9.81 | 10.24 | 10.38 | 10.93 | 11.59 | Site II |
| LYS-432 | 9.85 | 11.10 | 9.76 | 9.85 | 9.58 | 11.58 | |
| LYS-436 | 10.16 | >12.00 | 10.00 | 10.94 | 9.54 | >12.00 | |
| LYS-439 | 10.47 | 10.82 | 10.45 | 10.93 | 10.61 | 11.40 | |
| LYS-444 | 10.15 | 10.69 | 10.01 | 10.07 | 10.12 | 10.58 | |
| LYS-466 | 10.07 | 10.12 | 10.14 | >12.00 | 10.50 | 10.82 | |
| LYS-475 | 10.20 | 11.42 | 10.15 | 10.69 | 10.20 | 10.81 | |
| LYS-500 | 10.51 | 10.95 | 10.45 | 10.90 | 10.06 | >12.00 | |
| LYS-519 | 9.98 | 10.27 | 9.75 | 9.76 | 9.69 | 9.82 | |
| LYS-524 | 8.70 | 9.79 | 9.75 | 10.37 | 9.91 | 11.42 | |
| LYS-525 | 9.57 | 10.95 | 8.70 | 10.41 | 9.36 | 9.76 | |
| LYS-534 | 10.66 | >12.00 | 10.77 | >12.00 | 10.93 | >12.00 | |
| LYS-536 | 8.90 | 11.09 | 8.36 | 11.13 | 10.32 | 10.82 | |
| LYS-538 | 10.61 | 11.23 | 10.42 | 10.85 | 10.13 | 10.27 | |
| LYS-541 | 9.98 | 10.05 | 10.09 | 10.24 | 8.71 | 7.42 | |
| LYS-545 | 10.27 | 11.19 | 9.73 | 9.81 | 10.32 | >12.00 | |
| LYS-557 | 9.79 | >12.00 | 10.09 | 11.46 | 10.16 | 11.55 | |
| LYS-560 | 10.00 | 10.79 | 10.35 | 11.15 | 10.34 | 11.37 | |
| LYS-564 | 8.94 | 10.57 | 10.05 | 11.12 | 6.02 | 7.63 | Lowest pKa for 4L8U; |

| | | | | | | | |
|---|---|---|---|---|---|---|---|
| LYS-573 | 10.14 | 10.65 | 10.18 | 10.92 | 10.43 | 11.27 | Calculation from Previous Literature: pKa = 10.390 |
| LYS-574 | 10.11 | 11.18 | 10.27 | 10.73 | 10.25 | 11.08 | Calculation from Previous Literature: pKa = 10.890 |

* The calculation of 1AO6 was the arithmetic mean of two chains.

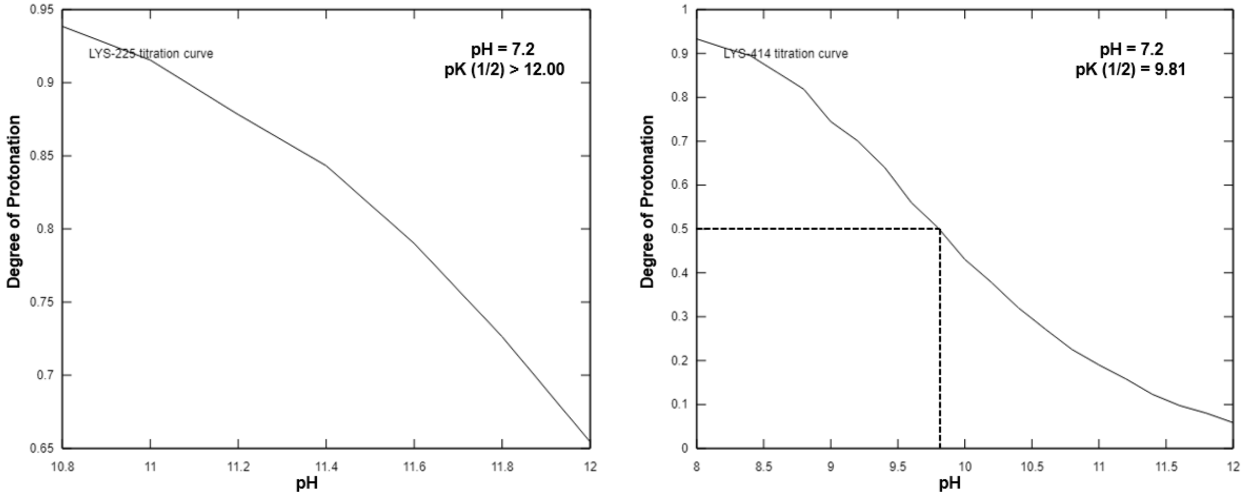


Figure S18 Predicted Titration Curve (PDB: 1BJ5) for pK (1/2) and degree of protonation of LYS-225 (Left) and LYS-414 (Right).

## S9. General Procedures to Prepare and Characterize HSA-RAEs Conjugates by Electrophoresis and Western Blots

### S9.1. Preparation of Stock Solutions for Conjugation

2 mM HSA stock solution was prepared by PBS buffer (0.1 M sodium phosphate, 0.15 M sodium chloride, pH = 7.2). 10 mM B-LYL1-4 and B-LYLG stock solutions were prepared in DMSO. 1 mM FC-LYL1 stock solution was prepared with PBS buffer. 10 mM FITC stock solution and FC-LYL1 stock solution was prepared by dissolving in DMSO.

### S9.2. General Procedures for Electrophoresis & Western Blot

Samples for electrophoresis were prepared by 4X LDS sampling buffer, which was premixed with 20% 2-mercapthanol. The mixture was briefly sedimented by centrifugation and heated for 5 minutes at 95 °C to fully break disulfide bonds and reduce protein. Electrophoresis was done by SDS-NuPage 4-12% gradient Bis-Tris acrylamide gels at constant voltage of 120V for 1 hour. Subsequently, the protein on the gel was transferred to nitrocellulose membrane within 1 hour at 100V in 4°C by wet transfer. The membrane was then blocked by BSA blocking solution (5% BSA, 0.05% Tween 20 in PBS buffer), followed by incubation of streptavidin-Alexa 647 conjugate for 1 hour at room temperature. The streptavidin-Alexa 647 solution was removed, and the membrane was incubated in PBS buffer in dark for 10 minutes. The fluorescence signal was monitored by gel imager station (Bio-rad) under Alexa 647 channel. Coomassie blue staining was done using Safeblue (Invitrogen) solution for one hour to visualize all protein fragment. The stained gel was soaked in DI water for overnight to de-stain background staining.

### S9.3. Preparation of HSA Conjugates using BLYL1-4 and BLYLG

To 200 μL PBS buffer (0.1M sodium phosphate, 0.15M sodium chloride, pH = 7.2) was added 2 μL HSA stock solution and 2 μL RAE stock solution to prepare conjugation solution containing 20 μM HSA and 100 μM RAE. The conjugate mixture was incubated

at room temperature for 1 hour. 10 μL conjugates were taken for electrophoresis and Western blot without further purification.

### S9.4. HSA/BLYL-1 Conjugation in the Presence of IVIG

To 193 μL PBS buffer (0.1 M sodium phosphate, 0.15 M sodium chloride, pH = 7.2) was added 1 μL HSA stock solution, 1 μL BLYL-1 stock solution and 5 μL 0.13 mM IVIG stock solution to prepare conjugation solution containing 10 μM HSA (0.67mg/mL) and 3.3 μM IVIG (0.5 mg/mL) and 50μM BLYL-1. Blank and comparative experiments used PBS to replace the portion of RAEs. The ratio of HSA and IVIG concentration resembles the composition of HSA and globulin in human serum. (Assuming serum albumin content in serum: 35mg/mL, globulin content: 28mg/mL).[2] The solution was incubated at room temperature for 1 hour. 10 μL conjugates were aliquoted for electrophoresis and electro-blotting without further purification (Figure S14).

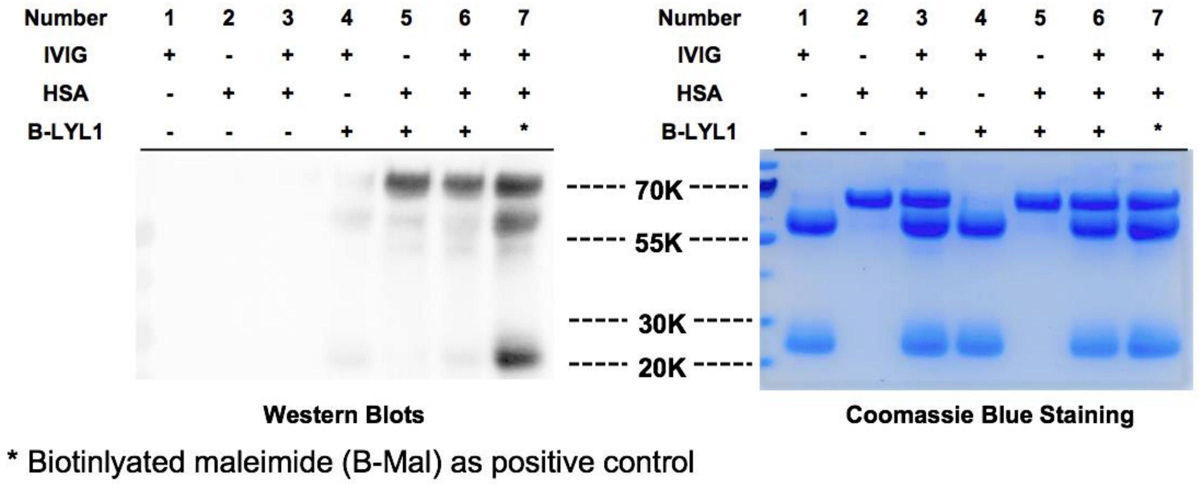


Figure S19 Electrophoresis and Western Blots for HSA Conjugation in the Presence of IVIG at RAE/HSA Molar Ratio at 1 : 5 under room temperature for 1 hour Under reducing condition, heavy chains and light chains of IVIG dissociated, resulting in the detection of two bands at 50kD and 25kD, respectively, by Western blot (Lane 1). In lane 7, as expected, HSA as well as both heavy and light chains of IVIG and HSA were found to be readily biotinylated by biotin-maleimide conjugate. In contrast, B-LYL1 was able to generate a strong biotin signal for HSA but not for IVIG.

### S9.5. BLYL-1 Conjugation to Serum-borne HSA

To 100 μL human serum was added 10 μL FC-LYL1 or 10 μL FC-Mal stock solution (1 mM). The albumin content in serum is presumed to be 600 μM for healthy donors. The

final concentrations of albumin and FC-labeled reactive probes in the reaction mixture are calculated to be 545 μM and 91 μM, respectively. The serum/ligands mixture was incubated at 37°C in dark. The conjugation reactions were setup every 3 days, and 14 conjugation reactions were setup in total for 3 weeks. The serum mixtures were subjected to electrophoresis and Coommassie Blue Staining without further treatment. Since albumin to probe ratio was about 6:1 and there are 7 known fatty acid binding sites on each albumin molecule, fatty acid binding site: probe ratio was over 42:1. We therefore believe significant portion of the reactive probes would be sequestered in the "non-productive" (no covalent ligation) fatty acid binding sites most of the time. As a result, unlike the result shown in Figures S11-13 and Tables S3 and 4 with fast ligation kinetics and good yield (in the setting of excess LYL-1 to albumin), here we observed slow reaction over days for both probes. Nonetheless, this experiment does confirm that site-specific ligation of albumin in the context of complex human serum can be achieved with LYL-1 probe, albeit taken days to complete.

**S9.6. One-pot HSA Dual-Modification by B-LYL1 and FC-Mal/B-Mal**

To PBS buffer (0.1 M sodium phosphate, 0.15 M sodium chloride, pH = 7.2) was added 1 μL HSA stock solution, 2 μL BLYL-1 stock solution and 5 μL FC-Mal or Mal-BT solution to prepare 200 μL conjugation solution containing 10 μM HSA. The molar ratio between HSA and B-LYL1 is 1 : 10, and the molar ratio between HSA and FC-Mal or B-Mal is 1 : 5. PBS buffer was used to replace corresponding composition for Blank and comparative experiments. The solution was incubated at room temperature for 1 hour in dark. 10 μL conjugates were taken for electrophoresis and Western blot analysis without further purification.

**S9.7 One-pot HSA Dual-Modification by B-LYL1 and FITC**

To PBS buffer (0.1 M sodium phosphate, 0.15 M sodium chloride, pH = 7.2 or 8.0) was added 1 μL HSA stock solution, 2 μL 10mM FITC stock solution and/or 2 μL 10 mM FC-LYL1 stock solution to make 200 μL conjugation solution containing 10 μM HSA. The molar ration between FITC/BLYL-1 and HSA is 10:1. The solution was incubated at

room temperature for 1 hour in dark. 10 μL conjugates were taken for electrophoresis and Western blot analysis without further purification.

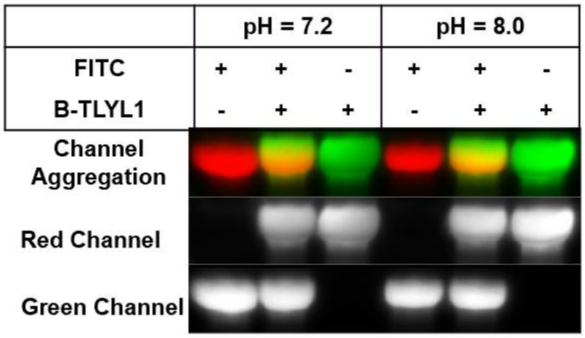


Figure S20 Conjugation with B-LYL1 and FITC under neutral (pH 7.2) and Alkaline (pH 8.0) in PBS buffer

### S9.8 One-pot HSA Dual-Modification by FC-LYL1 and Sulfo-NHS-Ester

To PBS buffer (0.1 M sodium phosphate, 0.15 M sodium chloride, pH = 7.2) was added 1 μL HSA stock solution, 2 μL 10mM FC-LYL1 DMSO stock solution. The molar ration between FITC/BLYL-1 and HSA is 10:1. Sulfo-NHS-Biotin solutions at various concentration were prepared by serial dilution using PBS buffer. The resulting solutions were added to HSA solution, which was incubated at room temperature for 1 hour in dark. 10 μL conjugates were taken for electrophoresis and Western blot analysis without further purification.

## S10. Characterization by MALDI-TOF

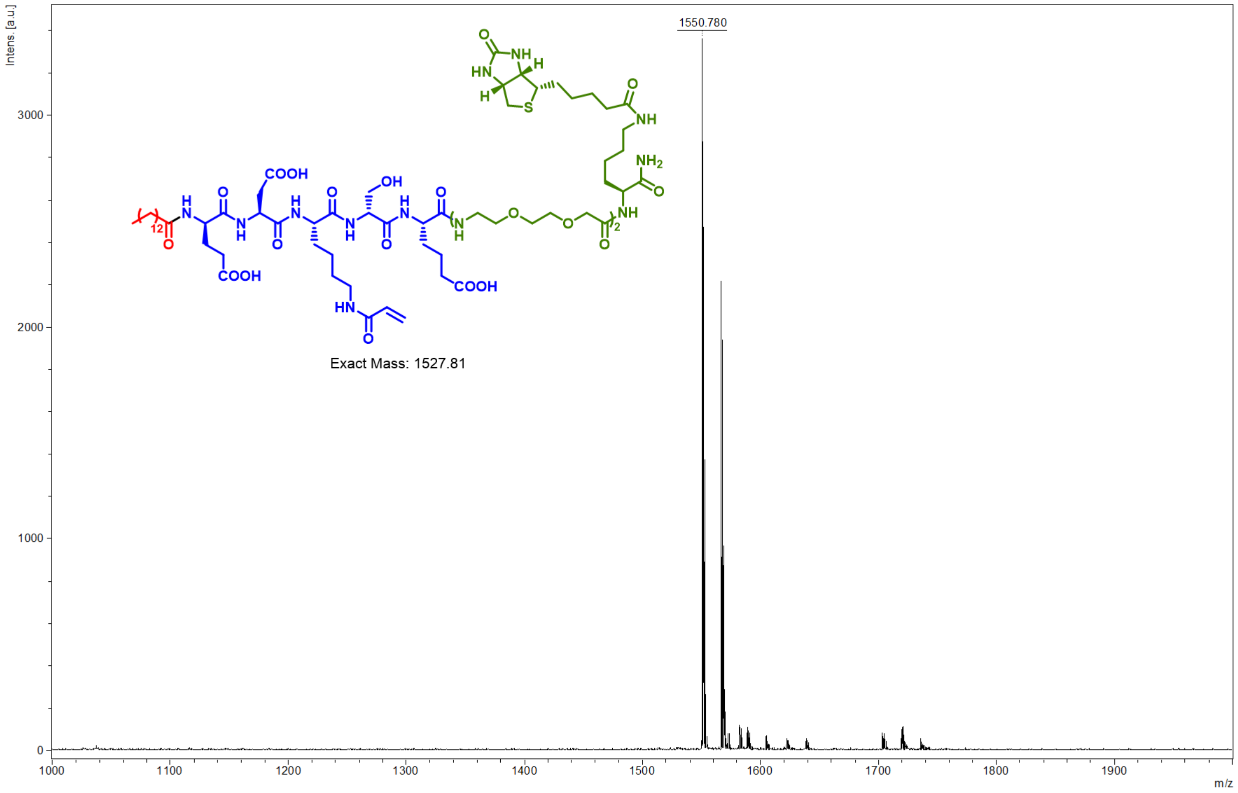


Figure M1 MALDI-TOF Mass Spectrometry for BLYL-1 (M + 23, M + 39)

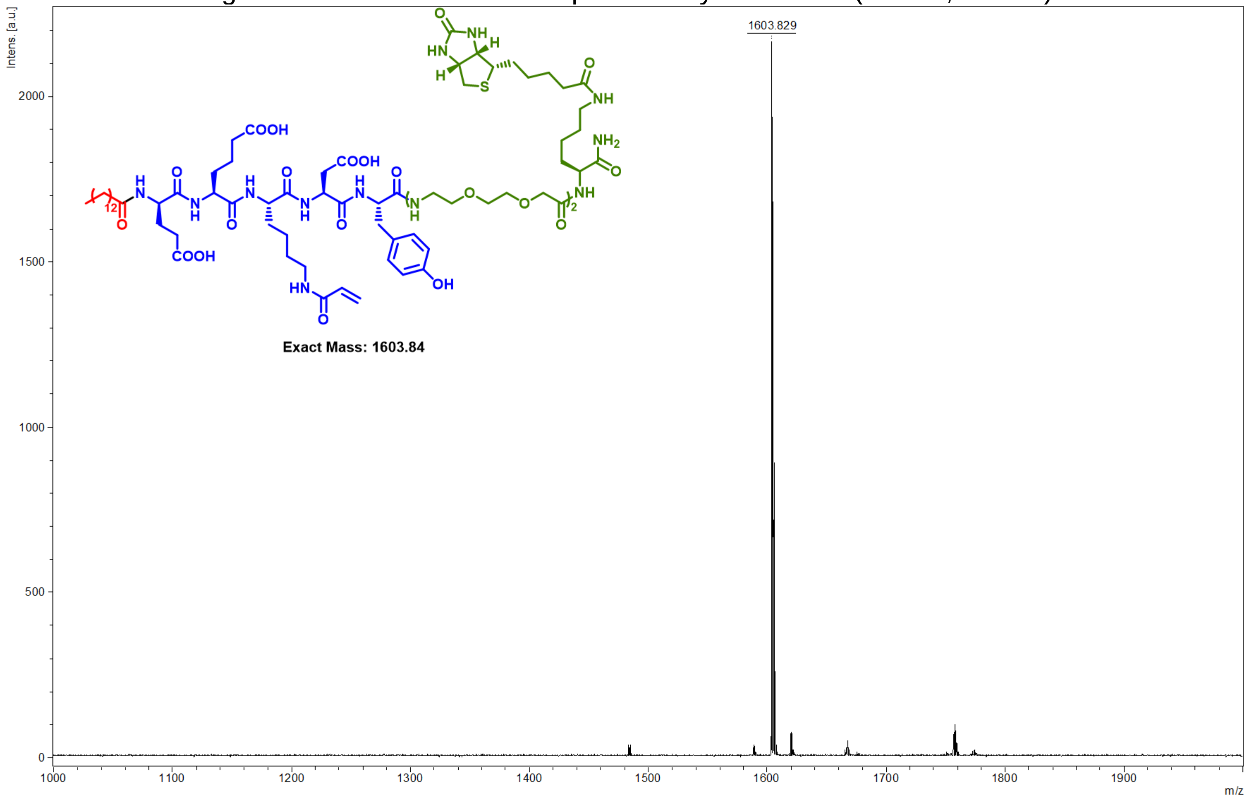


Figure M 2 MALDI-TOF Mass Spectrometry for BLYL-2 (Negative Mode)

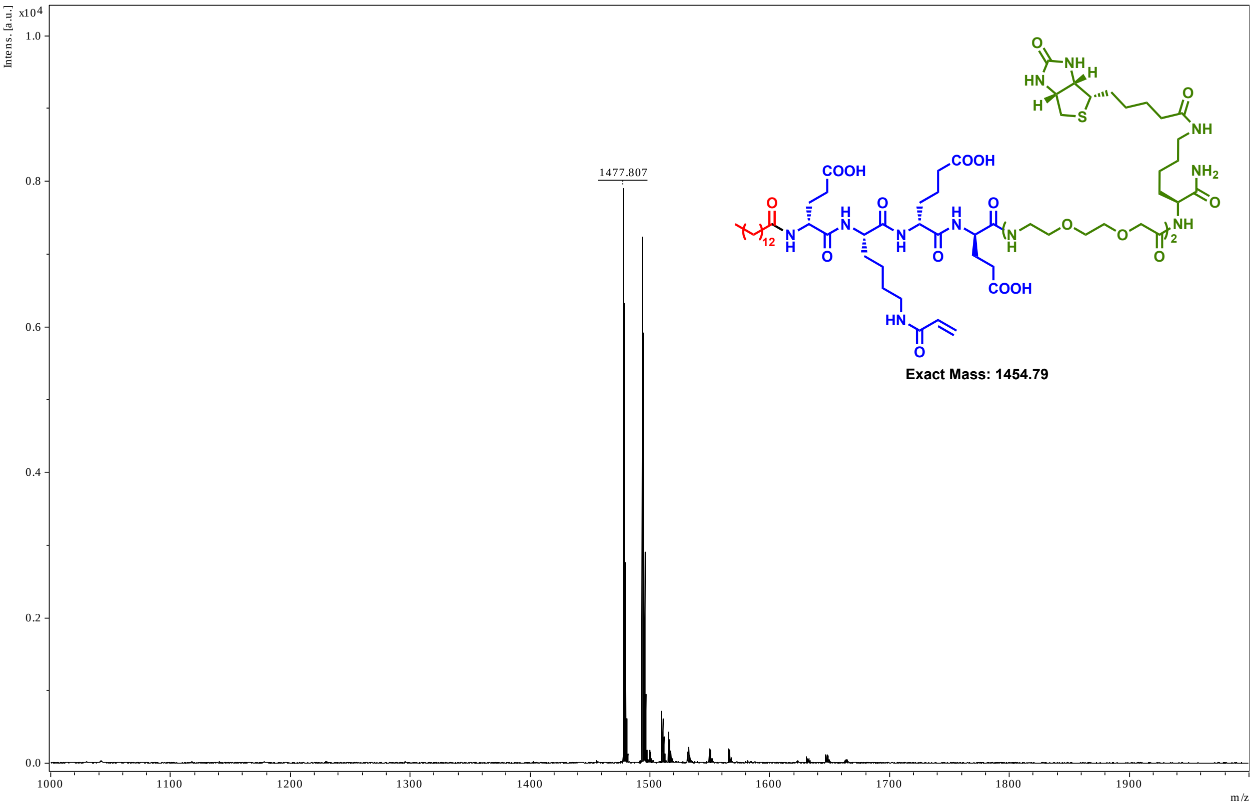


Figure M 3 MALDI-TOF Mass Spectrometry for BLYL-3 (M + 23, M + 39)

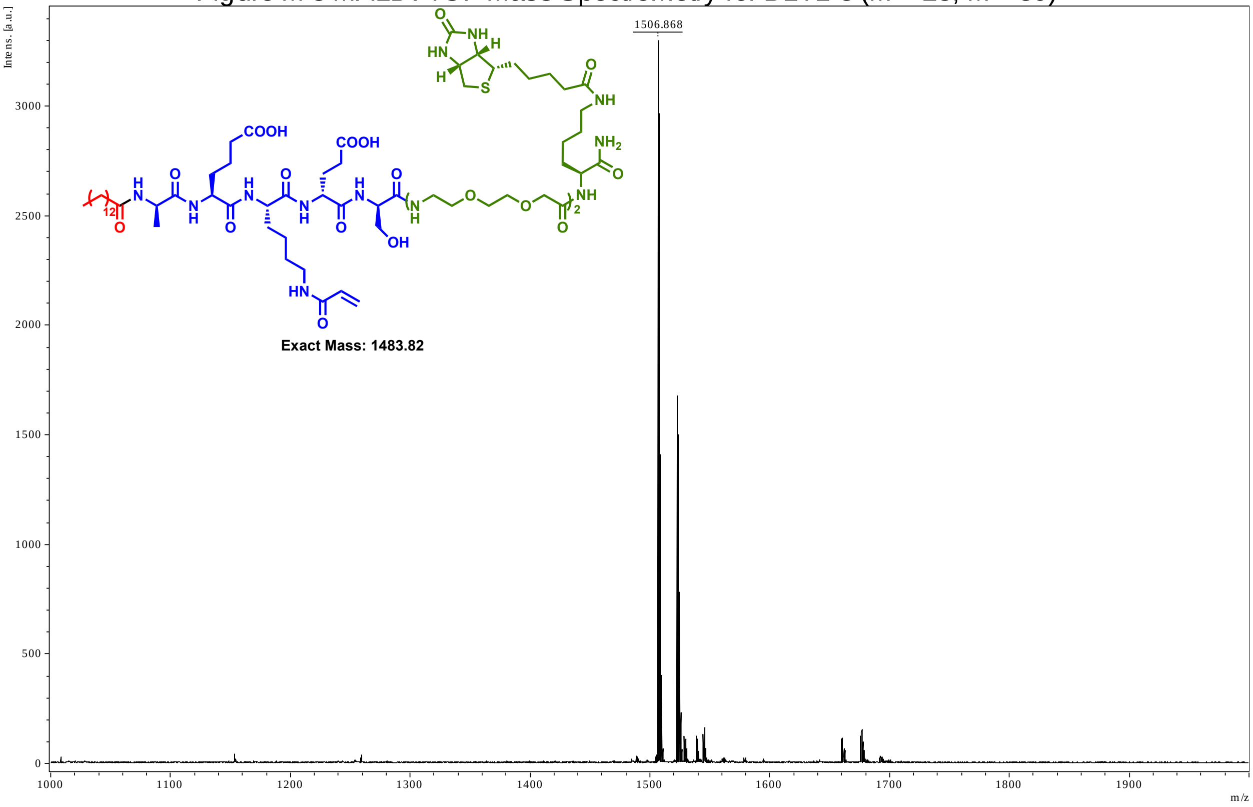


Figure M4 MALDI-TOF Mass Spectrometry for BLYL-4 (M+23 , M+39)

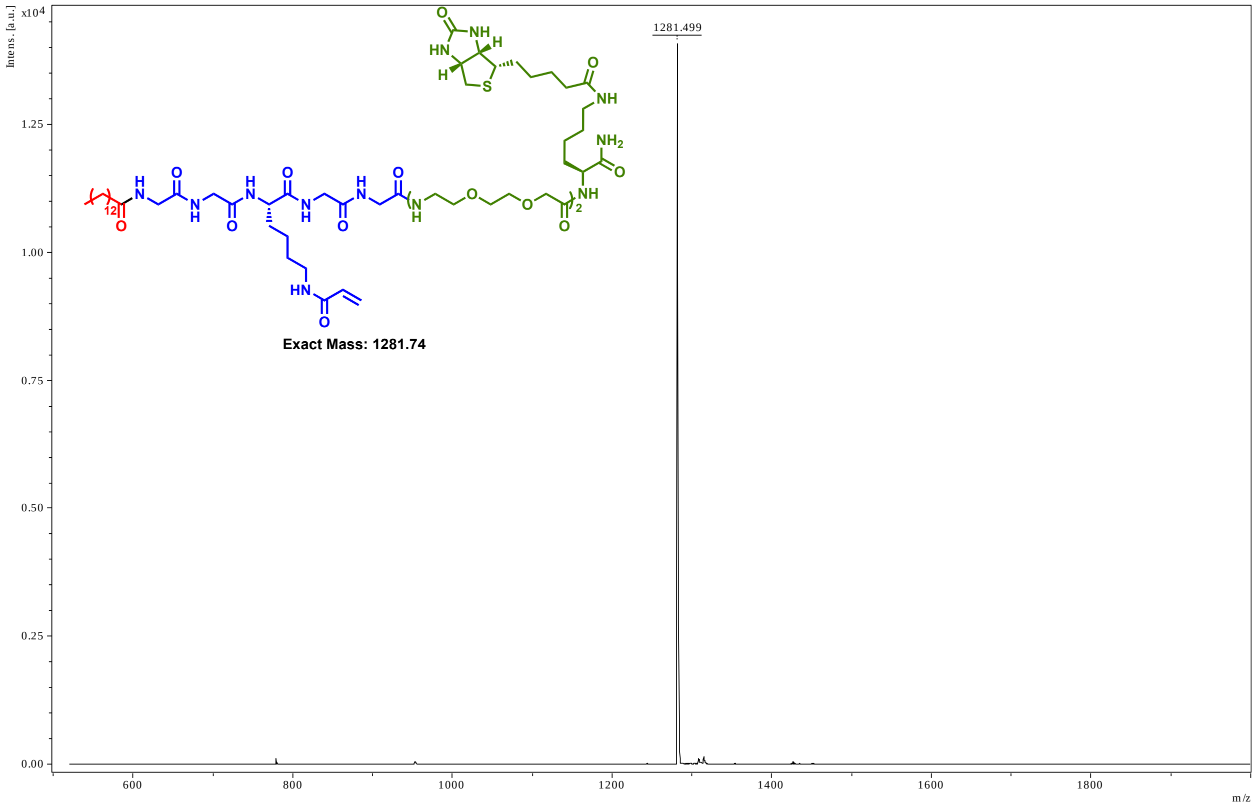


Figure M 5 MALDI-TOF Mass Spectrometry for BLYL-4

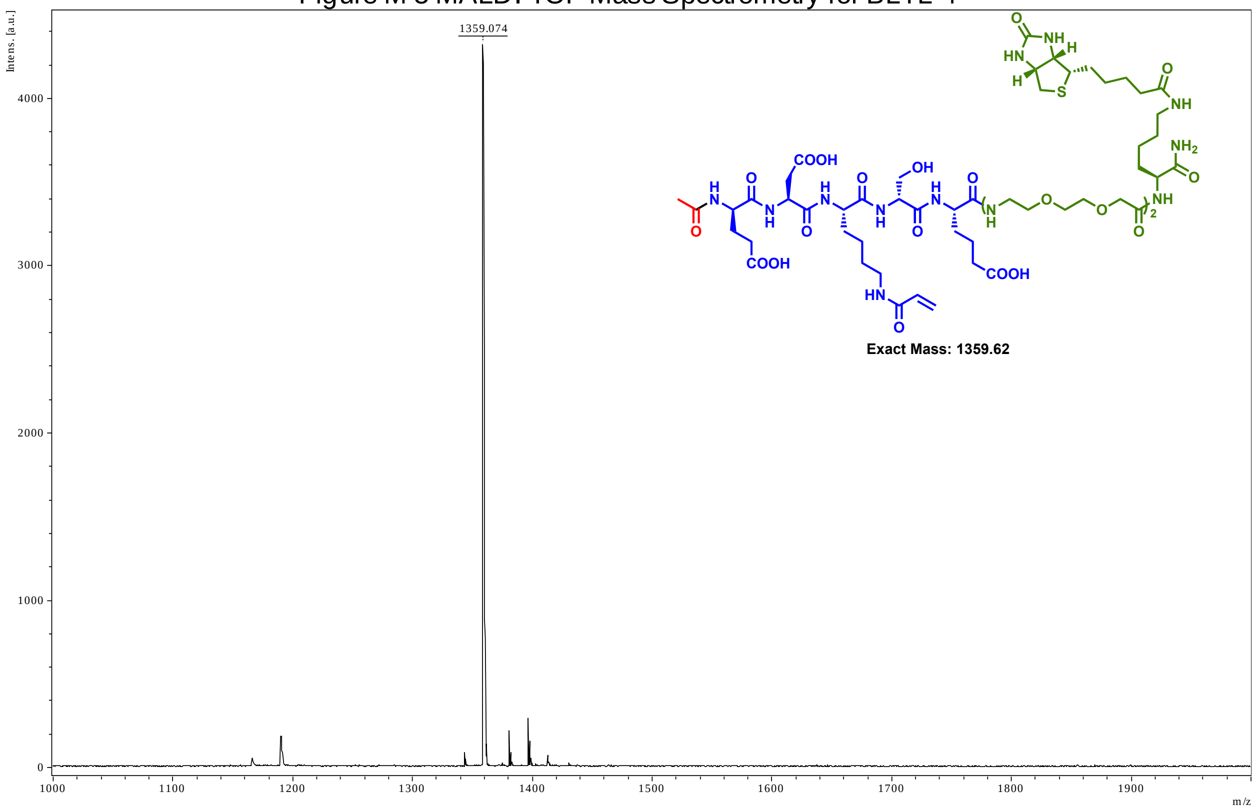


Figure M 6 MALDI-TOF Mass Spectrometry for Acrylated BLTL1 (Negative Mode)

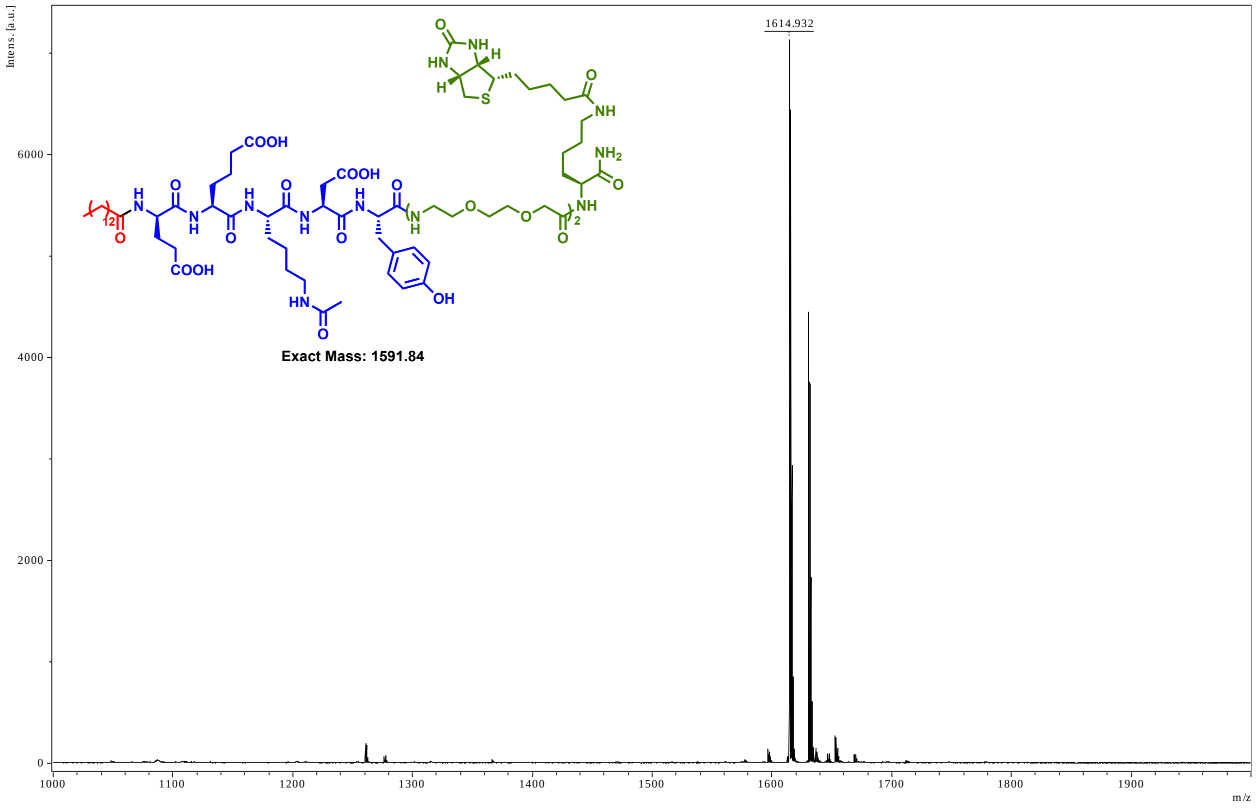


Figure M 7 MALDI-TOF Mass Spectrometry for NCB-LYL2 (M+23, M+39)

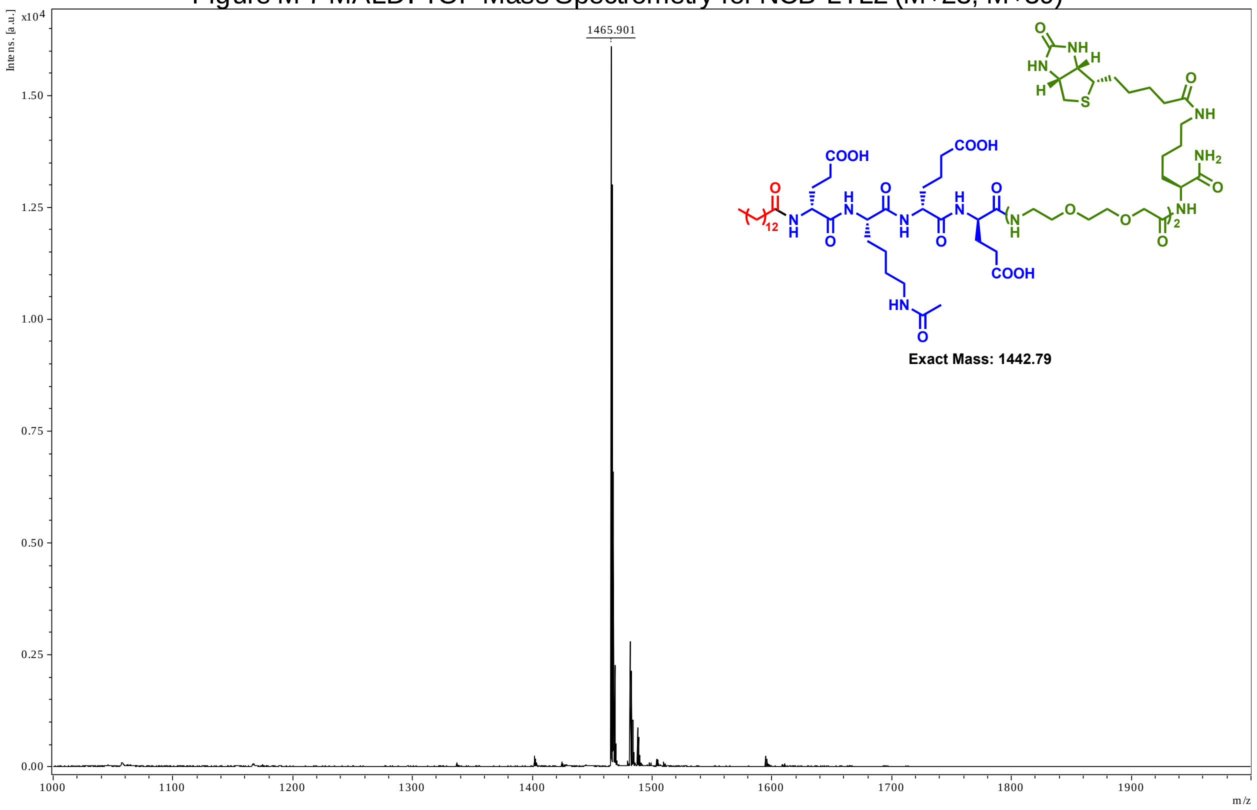


Figure M 8 MALDI-TOF Mass Spectrometry for NCB-LYL3 (M+23, M+39)

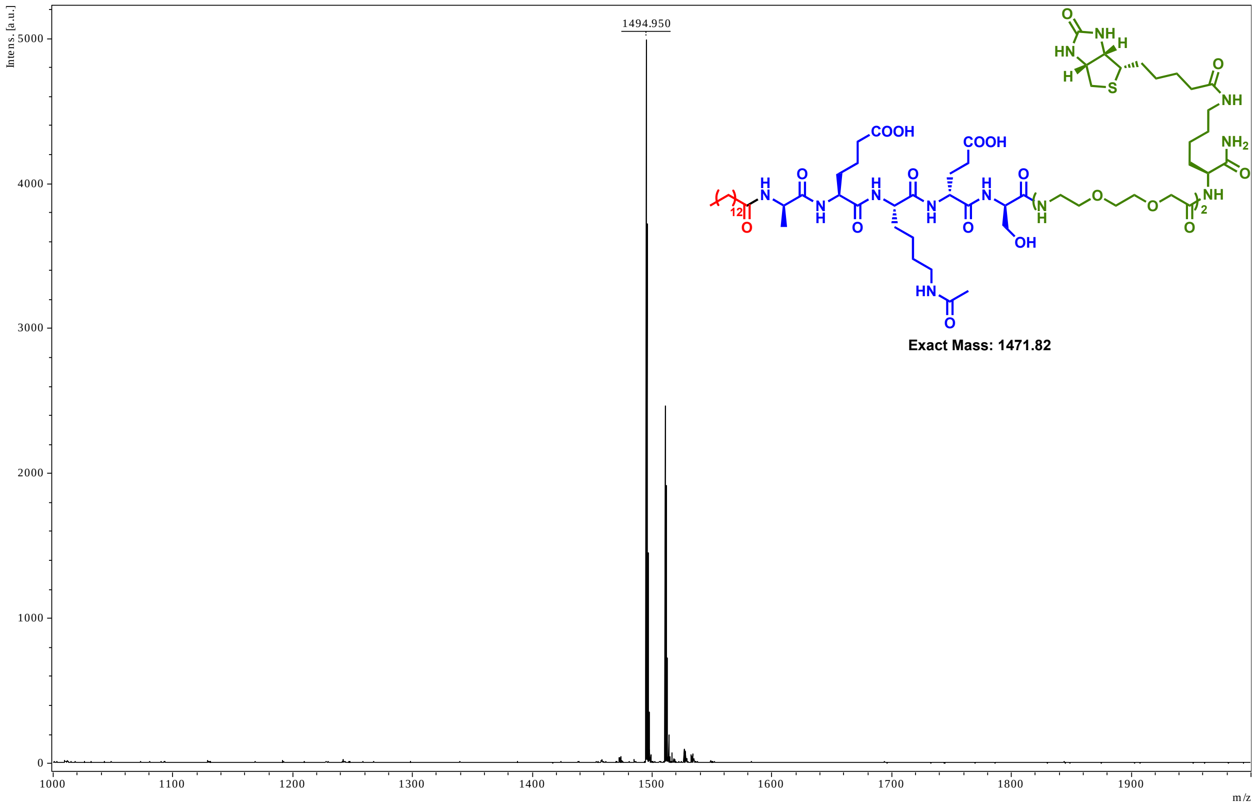


Figure M 9 MALDI-TOF Mass Spectrometry for NCB-LYL4 (M+23, M+39)

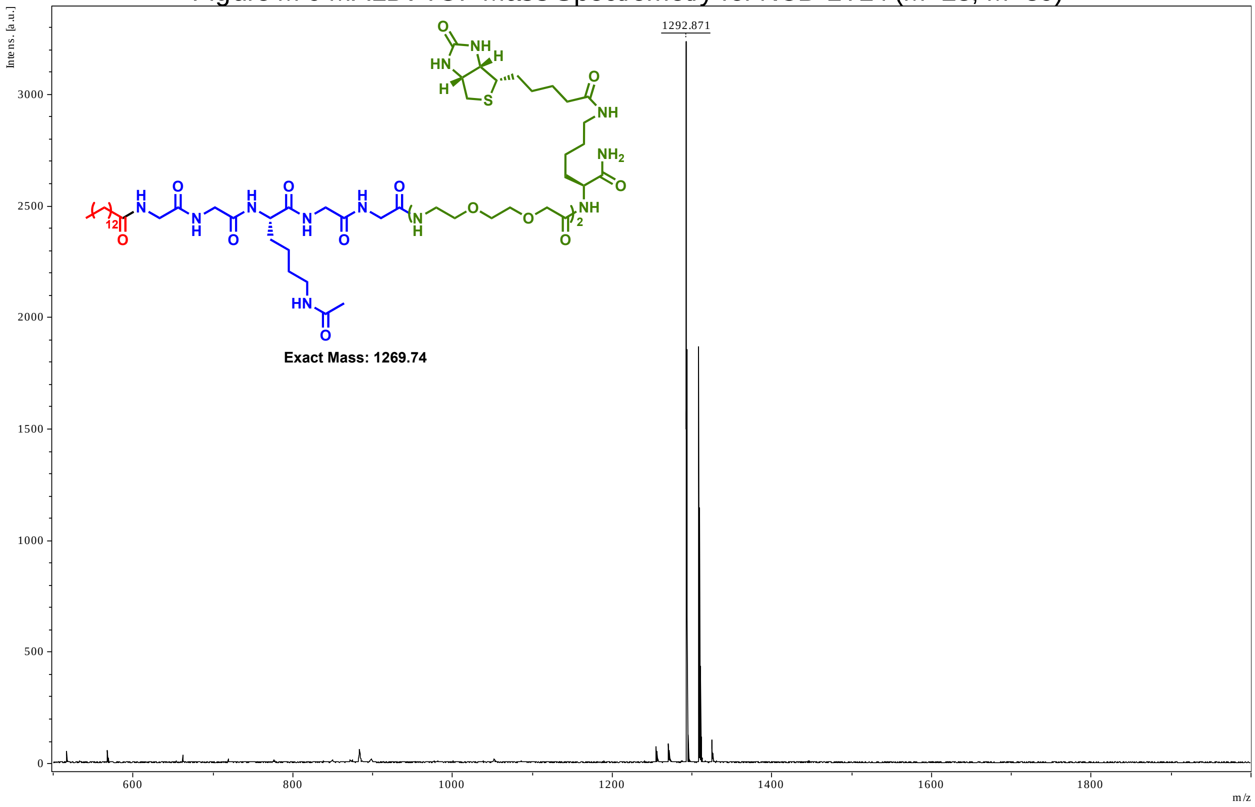


Figure M 10 MALDI-TOF Mass Spectrometry for NCB-LYLG (M+23, M+39)

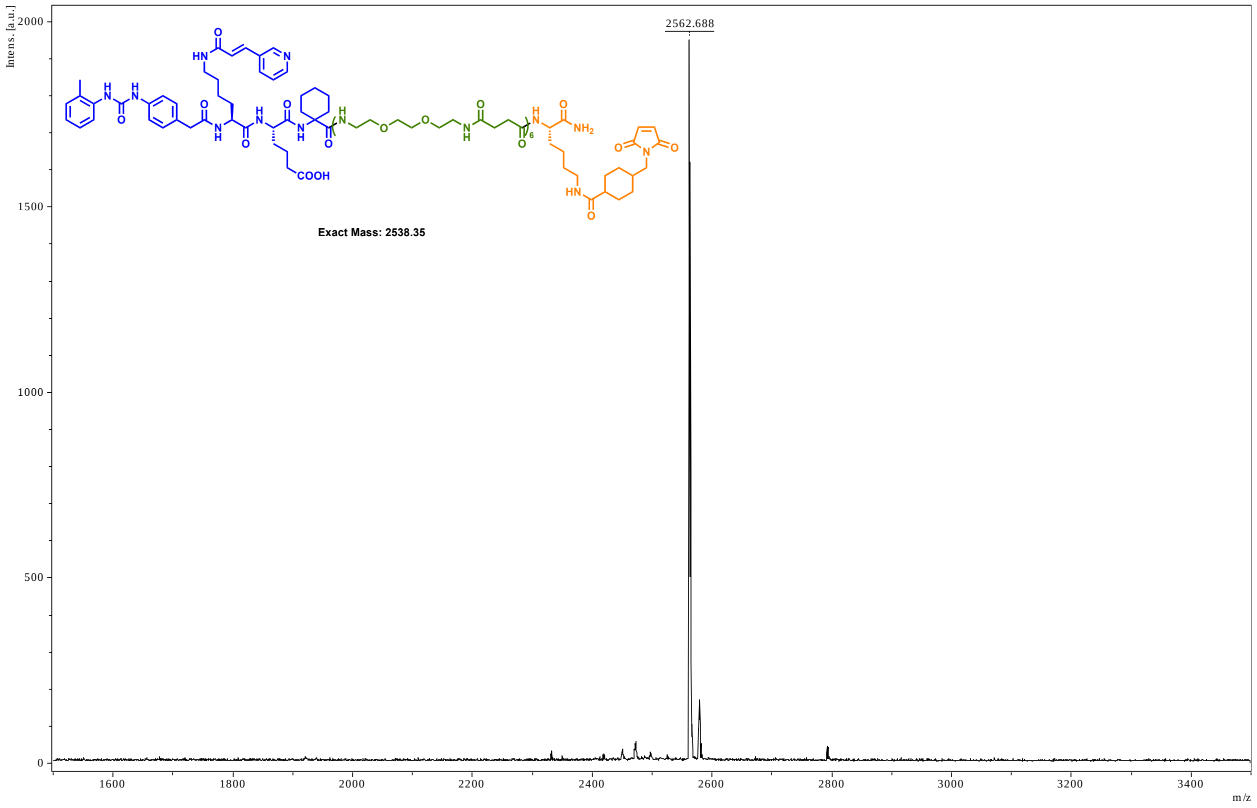


Figure M 11 MALDI-TOF Mass Spectrometry for LLP2A-Mal (M+23, M+39)

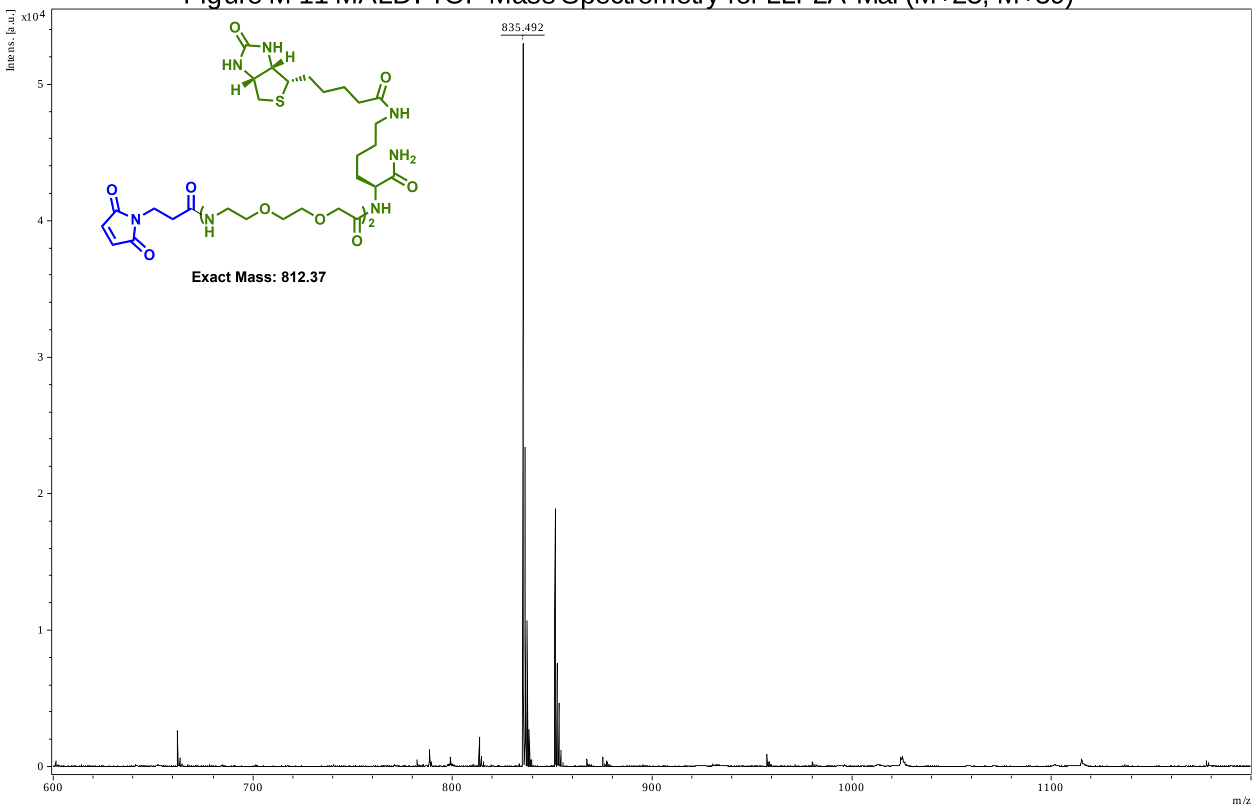


Figure M12 MALDI-TOF Mass Spectrometry for B-Mal (M+23, M+39)

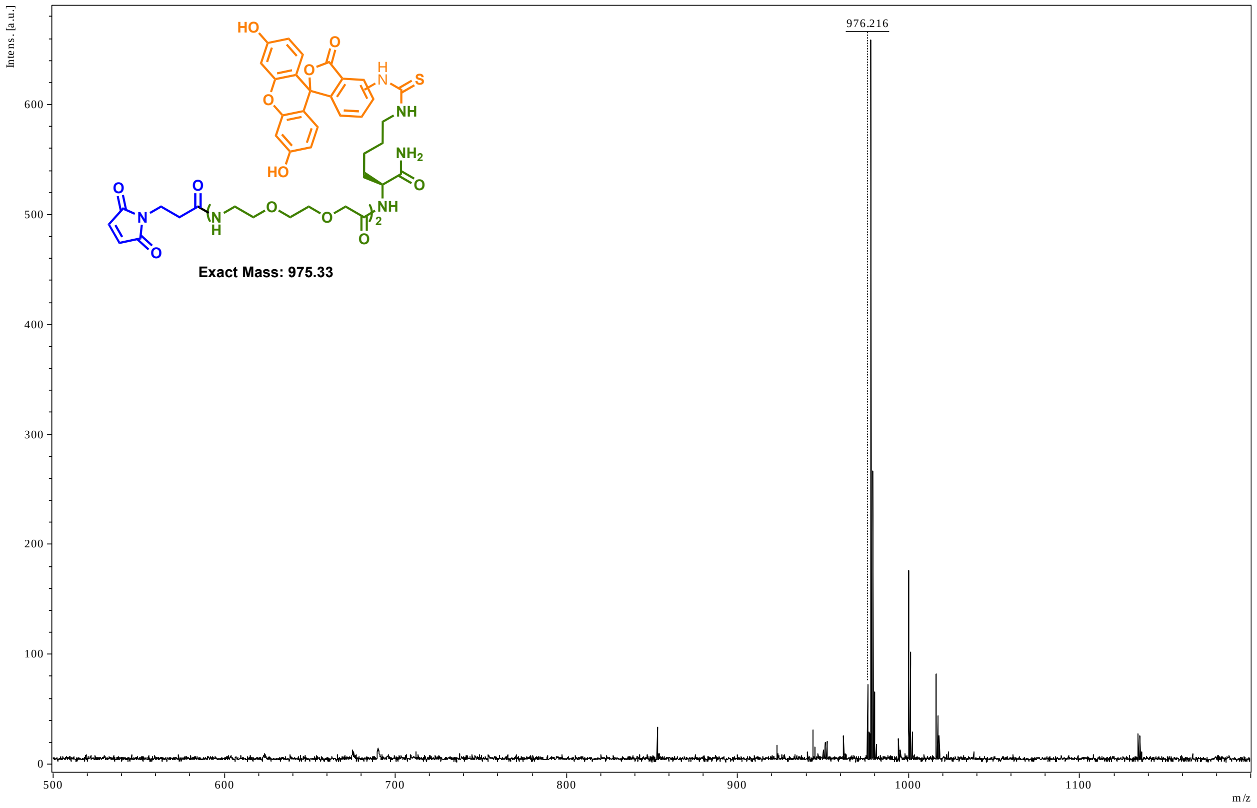


Figure M 13 MALDI-TOF Mass Spectrometry for FC-Mal

# Reference


1. Liu, R.; Marik, J.; Lam, K. S., A novel peptide-based encoding system for "one-bead one-compound" peptidomimetic and small molecule combinatorial libraries. *Journal of the American Chemical Society* **2002,** *124* (26), 7678-7680.
2. Leeman, M.; Choi, J.; Hansson, S.; Storm, M. U.; Nilsson, L., Proteins and antibodies in serum, plasma, and whole blood—size characterization using asymmetrical flow field-flow fractionation (AF4). *Analytical and bioanalytical chemistry* **2018,** *410* (20), 4867-4873.
3. Sugio, S.; Kashima, A.; Mochizuki, S.; Noda, M.; Kobayashi, K., Crystal structure of human serum albumin at 2.5 Å resolution. *Protein engineering* **1999,** *12* (6), 439-446.
4. Curry, S.; Mandelkow, H.; Brick, P.; Franks, N., Crystal structure of human serum albumin complexed with fatty acid reveals an asymmetric distribution of binding sites. *Nature structural biology* **1998,** *5* (9), 827-835.
5. Wang, Z.-m.; Ho, J. X.; Ruble, J. R.; Rose, J.; Rüker, F.; Ellenburg, M.; Murphy, R.; Click, J.; Soistman, E.; Wilkerson, L., Structural studies of several clinically important oncology drugs in complex with human serum albumin. *Biochimica et Biophysica Acta (BBA)-General Subjects* **2013,** *1830* (12), 5356-5374.
6. Matos, M. J.; Oliveira, B. L.; Martínez-Sáez, N.; Guerreiro, A.; Cal, P. M.; Bertoldo, J.; Maneiro, M.; Perkins, E.; Howard, J.; Deery, M. J., Chemo-and regioselective lysine modification on native proteins. *Journal of the American Chemical Society* **2018,** *140* (11), 4004-4017.
7. Liu, Q.; Simpson, D. C.; Gronert, S., The reactivity of human serum albumin toward trans-4-hydroxy-2-nonenal. *Journal of mass spectrometry* **2012,** *47* (4), 411-424.
